# Whole-Exome Sequencing Among Chinese Patients With Hereditary Diffuse Gastric Cancer

**DOI:** 10.1001/jamanetworkopen.2022.45836

**Published:** 2022-12-09

**Authors:** Ze-Xian Liu, Xiao-Long Zhang, Qi Zhao, Yungchang Chen, Hui Sheng, Cai-Yun He, Yu-Ting Sun, Ming-Yu Lai, Min-Qing Wu, Zhi-Xiang Zuo, Wei Wang, Zhi-Wei Zhou, Feng-Hua Wang, Yu-Hong Li, Rui-Hua Xu, Miao-Zhen Qiu

**Affiliations:** 1 State Key Laboratory of Oncology in South China, Collaborative Innovation Center for Cancer Medicine, Sun Yat-sen University Cancer Center, Guangzhou, People’s Republic of China; 2 Department of Medical Oncology, The First People’s Hospital of Foshan, Chancheng District, Foshan, People’s Republic of China; 3 Department of Molecular Diagnostics, Sun Yat-sen University Cancer Center, State Key Laboratory of Oncology in South China, Collaborative Innovation Center for Cancer Medicine, Guangzhou, People’s Republic of China; 4 Department of Medical Oncology, Sun Yat-sen University Cancer Center, State Key Laboratory of Oncology in South China, Collaborative Innovation Center for Cancer Medicine, Guangzhou, People’s Republic of China; 5 Department of Cancer Prevention, Sun Yat-sen University Cancer Center, Guangzhou, People’s Republic of China; 6 Department of Gastric Surgery, Sun Yat-sen University Cancer Center, State Key Laboratory of Oncology in South China, Collaborative Innovation Center for Cancer Medicine, Guangzhou, People’s Republic of China; 7 Research Unit of Precision Diagnosis and Treatment for Gastrointestinal Cancer, Chinese Academy of Medical Sciences, Guangzhou, People’s Republic of China

## Abstract

**Importance:**

The E-cadherin gene, *CDH1*, and the α-E-catenin gene, *CTNNA1*, were previously identified as hereditary diffuse gastric cancer (HDGC) susceptibility genes, explaining 25% to 50% of HDGC cases. The genetic basis underlying disease susceptibility in the remaining 50% to 75% of patients with HDGC is still unknown.

**Objective:**

To assess the incidence rate of *CDH1* germline alterations in HDGC, identify new susceptibility genes that can be used for screening of HDGC, and provide a genetic landscape for HDGC.

**Design, Setting, and Participants:**

This cohort study conducted retrospective whole-exome and targeted sequencing of 284 leukocyte samples and 186 paired tumor samples from Chinese patients with HDGC over a long follow-up period (median, 21.7 [range, 0.6-185.9] months). Among 10 431 patients diagnosed with gastric cancer between January 1, 2002, and August 31, 2018, 284 patients who met the criteria for HDGC were included. Data were analyzed from August 1 to 30, 2020.

**Main Outcomes and Measures:**

Incidence rate of *CDH1* germline alterations, identification of new HDGC susceptibility genes, and genetic landscape of HDGC.

**Results:**

Among 284 Chinese patients, 161 (56.7%) were female, and the median age was 35 (range, 20-75) years. The frequency of *CDH1* germline alterations was 2.8%, whereas the frequency of *CDH1* somatic alterations was 25.3%. The genes with the highest incidence (>10%) of private germline alterations (including insertions and deletions) in the HDGC cohort were *MUC4*, *ABCA13*, *ZNF469*, *FCGBP*, *IGFN1*, *RNF213*, and *SSPO*, whereas previously reported germline alterations of *CTNNA1*, *BRCA2*, *STK11*, *PRSS1*, *ATM*, *MSR1*, *PALB2*, *BRCA1*, and *RAD51C* were observed at low frequencies (median, 4 [range, 1-12] cases). Furthermore, enrichment of the somatic variant signature of exposure to aflatoxin suggested potential interaction between genetics and environment in HDGC. Double-hit events in genes such as *CACNA1D* were observed, which suggested that these events might serve as important mechanisms for HDGC tumorigenesis. In addition, germline variants of *FSIP2*, *HSPG2*, and *NCKAP5* and somatic alterations of *FGFR3*, *ASPSCR1*, *CIC*, *DGCR8*, and *LZTR1* were associated with poor overall survival among patients with HDGC.

**Conclusions and Relevance:**

This study provided a genetic landscape for HDGC. The study’s findings challenged the previously reported high germline alteration rate of *CDH1* in HDGC and identified new potential susceptibility genes. Analyses of variant signatures and double-hit events revealed potentially important mechanisms for HDGC tumorigenesis. Findings from the present study may provide helpful information for further investigations of HDGC.

## Key Points

**Questions:**

What is the incidence rate of germline alterations in *CDH1*, which has been reported as a susceptibility gene present in 25% to 50% of patients with hereditary diffuse gastric cancer (HDGC), and is there a genetic basis underlying disease susceptibility in the remaining 50% to 75% of patients with HDGC?

**Findings:**

In this cohort study of 284 Chinese patients with HDGC, the frequency of *CDH1* germline alterations was low (2.8%), and germline alterations, insertions, and deletions were most frequently found in *MUC4*, *ABCA13*, *ZNF469*, *FCGBP*, *IGFN1*, *RNF213*, and *SSPO*. Double-hit events in genes such as *CACNA1D* were observed among patients with HDGC.

**Meaning:**

This study’s findings challenge the previously reported high frequency of *CDH1* germline alterations in HDGC and suggest that double-hit events may serve as important mechanisms for HDGC tumorigenesis; the study also provided a genetic landscape and identified new susceptibility genes for HDGC.

## Teaser

This cohort study uses whole-exome and targeted sequencing to assess the incidence rate of *CDH1* germline alterations in hereditary diffuse gastric cancer, provide a genetic landscape for the disease, and identify new susceptibility genes among Chinese patients with hereditary diffuse gastric cancer.

## Introduction

Gastric cancer (GC) is the fifth most common cancer and the third most common cause of cancer-related death worldwide.^1^ The disease is particularly common in East Asia.^2^ Although most GCs occur sporadically, aggregation within families occurs in approximately 10% of cases.^3^ It has now been established that 3% to 5% of GCs are associated with inherited GC predisposition syndromes, including hereditary diffuse GC (HDGC), gastric adenocarcinoma and proximal polyposis of the stomach, and familial intestinal GC.^4,5,6^

Hereditary diffuse GC is thought to be associated with inherited genetic susceptibility. The E-cadherin gene, *CDH1*, was previously identified as an HDGC susceptibility gene, explaining 25% to 50% of HDGC cases.^7,8,9^ Carriage of the abnormal *CDH1* gene confers a greater than 80% lifetime risk of developing GC.^5^ Alterations in the α-E-catenin gene, *CTNNA1*, have been identified as additional genetic factors associated with HDGC.^10^ In a large HDGC pedigree, a germline truncating allele of *CTNNA1* was present in 2 family members with invasive diffuse GC and 4 family members in which intramucosal signet ring cells were detected as part of endoscopic surveillance.^10^ Furthermore, increased risk of GC is associated with other previously reported HDGC susceptibility genes, including *BRCA1*, *MLH1*, *MSH2*, *MSH6*, *PMS2*, and *PALB2*.^11^ However, the alteration rate of *CDH1* in East Asian patients and the genetic basis underlying susceptibility in the remaining 50% to 75% of patients with HDGC are still unknown.

Advances in sequencing technology have led to pioneering studies profiling the genomic landscape of diffuse-type and non–diffuse-type GCs.^12,13,14,15,16,17^ The Cancer Genome Atlas (TCGA) project has further provided multinomics-based molecular characterization of gastric adenocarcinoma.^14^ Roberts et al^18^ defined the genetic heterogeneity of familial pancreatic cancer through whole-genome sequencing. The diagnosis of HDGC is based on clinical criteria, including age at diagnosis, family history, and Lauren classification,^3^ but the rationale for including individuals diagnosed with diffuse GC before age 40 years who do not have a family history of the disease deserves further consideration. Thus, characterization of the molecular portrait is needed to provide further understanding of the tumorigenesis of clinically defined HDGC. This cohort study aimed to assess the incidence rates of *CDH1* germline alterations in HDGC, identify new HDGC susceptibility genes, and provide a genetic landscape for HDGC.

## Methods

This retrospective cohort study was performed in accordance with the Declaration of Helsinki^19^ and was approved by the ethics committee of Sun Yat-sen University Cancer Center. All participants provided consent to report and publish their individual patient data. This study followed the Strengthening the Reporting of Observational Studies in Epidemiology (STROBE) reporting guideline for cohort studies.

We screened the clinical data of 10 431 patients who were diagnosed with GC at Sun Yat-sen University Cancer Center between January 1, 2002, and August 31, 2018 (eFigure 1 in Supplement 1). Data were analyzed from August 1 to 30, 2020. Patients were included if they met at least 1 of 4 criteria for HDGC: (1) a diagnosis of 2 or more cases of GC or 1 confirmed case of diffuse GC in first- or second-degree relatives before age 50 years; (2) 3 or more confirmed cases of diffuse GC among first- or second-degree relatives, independent of age at onset; (3) diagnosis of diffuse GC before age 40 years and without a family history of diffuse GC; or (4) personal or family history of diffuse GC and lobular breast cancer, 1 of which was diagnosed before age 50 years. Overall, 542 of 10 431 patients (5.2%) from Sun Yat-sen University Cancer Center met the criteria for HDGC; of those, 177 patients with insufficient leukocyte samples and 83 patients without detailed follow-up data were excluded. The remaining 282 patients with HDGC were included, and 2 patients with HDGC from The First People’s Hospital of Foshan were added to the sample, for a total of 284 participants.

Whole-exome sequencing was performed on 284 HDGC leukocyte samples and 186 paired tumor samples (eTable 4 in Supplement 2). Because HDGC is a rare disease, the analysis of germline alterations focused on private variants. Private variants were defined as (1) nonsense alterations, splice-site alterations, and frameshift insertions and deletions (indels); (2) variants heterozygous in the germline; (3) variants with less than 0.5% minor allele frequency in the 1000 Genomes Project^20^ database or the Chinese Millionome Database^21^; (4) variants present in only 1 patient; (5) variants with a mappability score greater than 0.5; and (6) variants with no additional genomic locus based on queries in the BLAT alignment tool.^22^ Given that most of the known high-penetrance disease–associated variants are located in the coding regions, only genetic alterations located in these regions were considered.

The MutSigCV algorithm, version 1.41,^23^ was used to detect significantly altered genes (with significance set at *Q *< .05). Somatic copy number alterations (SCNAs) were detected using the Control-FREEC algorithm, version 11.1.^24^ The GISTIC2.0 algorithm^25^ was used to identify recurrently amplified or deleted genomic regions in the HDGC cohort. G scores were calculated for sequencing regions based on the frequency and amplitude of amplification or deletions in each gene. A double-hit event was defined as a heterozygous germline alteration that became homozygous and may expand the impact of germline alterations and potentially be associated with tumorigenesis. Additional details on target capture sequencing; detection of somatic single-nucleotide variants (SSNVs), indels, and SCNAs; double-hit event analysis; and drug target analysis are provided in the eMethods in Supplement 1.

### Statistical Analysis

For comparison between categorical variables, 2-sided *P* values were calculated using a Fisher exact or χ^2^ test. For multiple testing corrections, the false discovery rate was calculated using the Benjamini-Hochberg procedure. Survival function estimation was performed using Kaplan-Meier estimates and a log-rank test. All statistical analyses were performed using R software, version 3.5.0 (R Foundation for Statistical Computing). Statistical significance was set at 2-sided *P* < .05.

## Results

Among 284 Chinese patients, 161 (56.7%) were female, and the median age was 35 (range, 20-75) years. Most patients had stage III or stage IV GC (190 patients [66.9%]) and were diagnosed with diffuse GC before age 40 years, with no family history of diffuse GC (254 patients [89.4%]). Median follow-up was 21.7 (range, 0.6-185.9) months. Only 8 patients (2.8%) had positive results for Epstein-Barr virus infection, which was lower than previously reported infection rates among patients with GC.^26^ The 5-year survival rate was 61.4% (95% CI, 53.2%-68.6%), which was higher than the survival rate of 44.1% reported in a previous study of patients with sporadic diffuse GC.^27^ Additional clinicopathological characteristics of enrolled patients are shown in the Table, and further details about the cohort and exome sequencing of patient samples are provided in the eResults in Supplement 1.

**Table.** Clinicopathological Characteristics of Patients With Hereditary Diffuse Gastric Cancer Included in Sequenced Blood or Tumor Sample Analysis

Characteristic	Patients, No. (%)	
	Blood sample analysis (n = 284)	Tumor sample analysis (n = 186)
Age, y		
<40	267 (94.0)	178 (95.7)
≥40	17 (6.0)	8 (4.3)
Sex		
Male	123 (43.3)	82 (44.1)
Female	161 (56.7)	104 (55.9)
Family history of diffuse GC		
Yes	30 (10.6)	15 (8.1)
No	254 (89.4)	171 (91.9)
Definition of HDGC		
Condition 1^a^	23 (8.1)	13 (7.0)
Condition 2^b^	7 (2.5)	2 (1.1)
Condition 3^c^	254 (89.4)	171 (91.9)
Condition 4^d^	0	0
AJCC tumor category^e^		
T1 or T2	65 (22.9)	39 (21.0)
T3 or T4	188 (66.2)	146 (78.5)
Unknown	31 (10.9)	1 (0.5)
AJCC node category^e^		
N0 or N1	91 (32.0)	55 (29.6)
N2 or N3	162 (57.0)	130 (69.9)
Unknown	31 (10.9)	1 (0.5)
AJCC metastasis category^e^		
M0	201 (70.8)	146 (78.5)
M1	83 (29.2)	40 (21.5)
AJCC cancer stage^e^		
I or II	94 (33.1)	57 (30.6)
III or IV	190 (66.9)	129 (69.4)
Differentiation status		
Moderate	14 (4.9)	8 (4.3)
Poor	264 (93.0)	172 (92.5)
Unknown	6 (2.1)	6 (3.2)
EBV status		
Positive	8 (2.8)	5 (2.7)
Negative	209 (73.6)	148 (79.6)
Unknown	63 (22.2)	33 (17.7)
*ERBB2* (formerly *HER2 or HER2/neu*) status		
Positive	6 (2.1)	3 (1.6)
Negative	215 (75.7)	155 (83.3)
Unknown	63 (22.2)	28 (15.1)
Relapse		
Yes	46 (16.2)	32 (17.2)
No	178 (62.7)	128 (68.8)
Unknown	60 (21.1)	26 (14.0)

Abbreviations: AJCC, American Joint Committee on Cancer; EBV, Epstein-Barr virus; GC, gastric cancer; HDGC, hereditary diffuse gastric cancer.

^a^ Condition 1 was defined as 2 or more cases of GC and 1 confirmed case of diffuse GC before age 50 years.

^b^ Condition 2 was defined as 3 or more cases of confirmed diffuse GC in first-degree or second-degree relatives, independent of age at onset.

^c^ Condition 3 was defined as diffuse GC before age 40 years without a family history of diffuse GC.

^d^ Condition 4 was defined as a personal or family history of diffuse GC and lobular breast cancer, one of which was diagnosed before age 50 years.

^e^ Based on the AJCC Cancer Staging Manual, Seventh edition.

### Germline Variant Profiling

**Figure 1.**
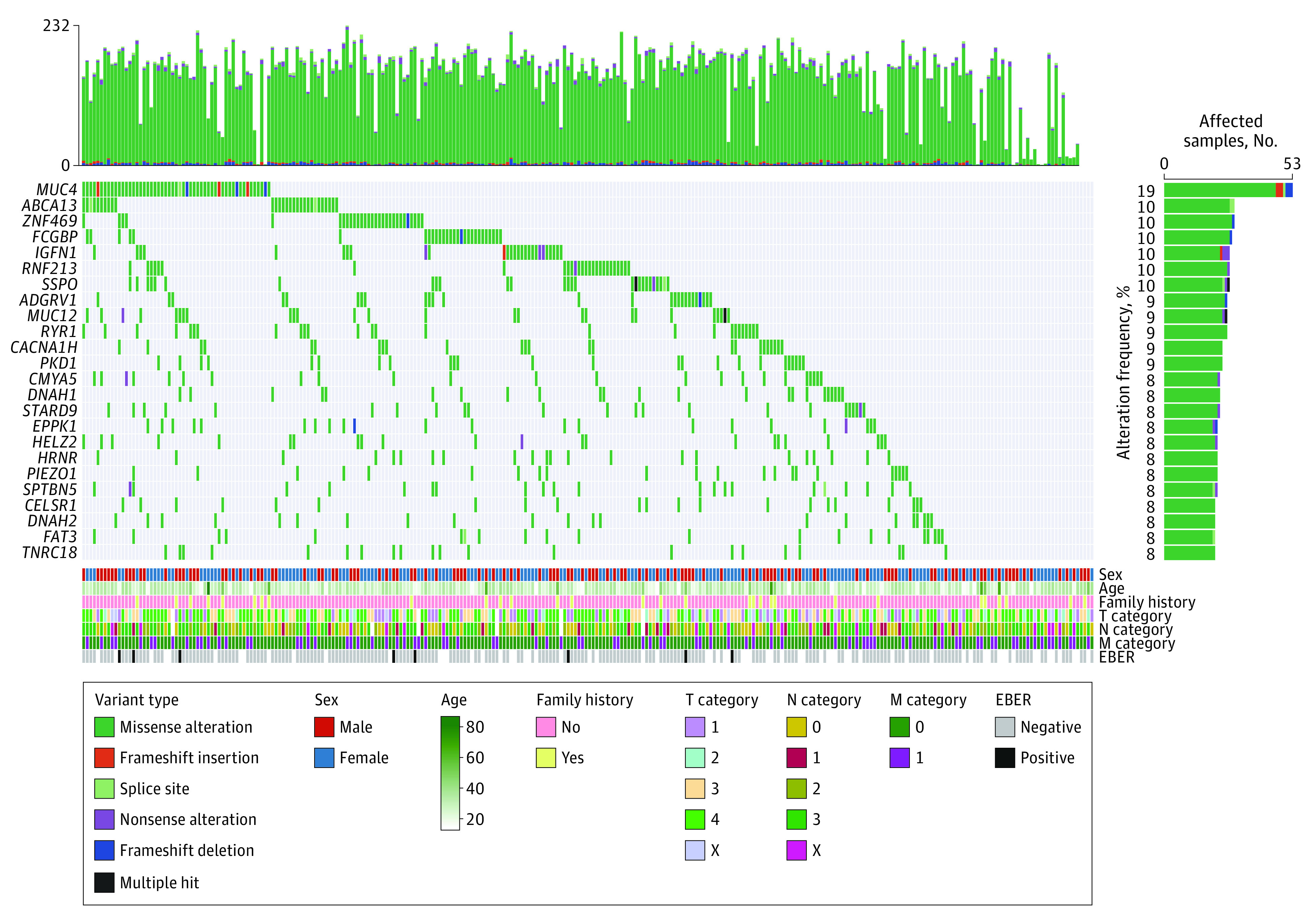
Germline Alteration Landscape of Hereditary Diffuse Gastric Cancer Oncoplot showing the germline alteration landscape among 284 Chinese patients with hereditary diffuse gastric cancer. The top panel shows the total of all nonsilent germline alterations for each sample. The middle panel shows the alteration details; only genes with alteration frequencies of 8% or greater are shown. The bottom panel shows clinical information. Cancer staging was based on the *AJCC Cancer Staging Manual, 7th edition*. EBER indicates Epstein-Barr virus–encoded small RNAs.

**Figure 2.**
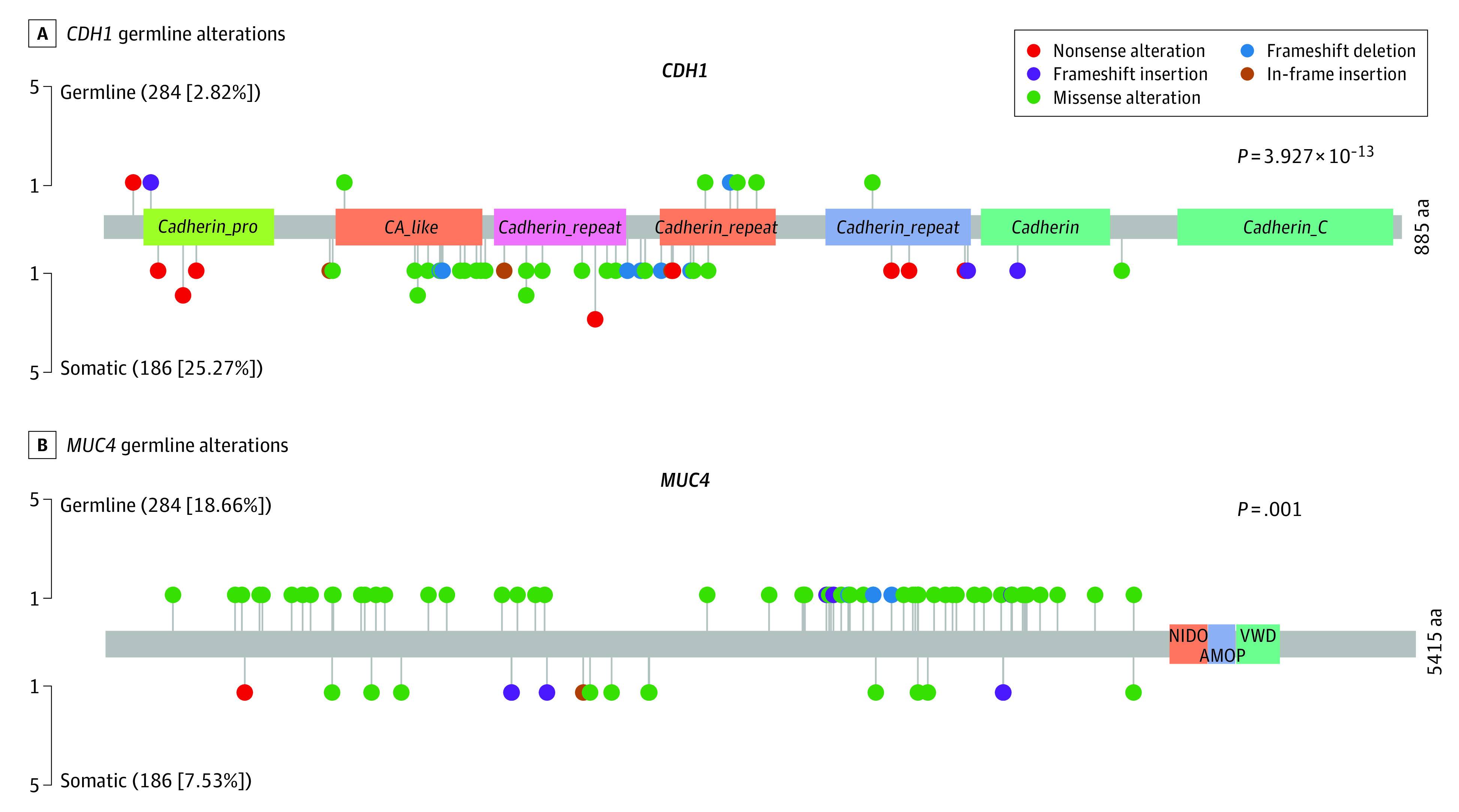
Germline Alterations of *CDH1* and *MUC4*

Based on the identified private germline variants (Figure 1), we examined genes potentially associated with susceptibility to HDGC (variant validation is shown in eTable 5 in Supplement 2). A previous study reported that 40% of HDGC cases had *CDH1* germline alterations.^28^ However, we detected *CDH1* germline missense alterations and indels in only 8 of 284 HDGC cases (2.8%) (Figure 2A), and all of the 6 missense alterations and 2 indels had not been previously reported. Even in patients with a family history of diffuse GC in this HDGC cohort, the frequency of *CDH1* germline alterations was still low (3.3%). The low frequency of *CDH1* germline variants suggested different genetic backgrounds and HDGC carcinogenesis mechanisms in East Asian populations. Furthermore, previous studies proposed that *CTNNA1*, *BRCA2*, *STK11*, SD*HB*, *PRSS1*, *ATM*, *MSR1*, *PALB2*, *BRCA1*, and *RAD51C* may be associated with susceptibility to non–*CDH1*-variant HDGC.^28,29^ In our study, germline single-nucleotide polymorphisms and indels of *CTNNA1* were observed in 2 HDGC cases, *BRCA2* in 12 cases, *STK11* in 2 cases, *PRSS1* in 2 cases, *ATM* in 5 cases, *MSR1* in 4 cases, *PALB2* in 6 cases, *BRCA1* in 6 cases, and *RAD51C* in 1 case. No alteration was found in SD*HB.* In addition, the observed germline single-nucleotide polymorphisms and indels in these genes were different from those reported in previous studies.^28,29^ The genes with the highest incidence rates (>10%) of private germline variants in the HDGC cohort were *MUC4* (19%), *ABCA13* (10%), *ZNF469* (10%), *FCGBP* (10%), *IGFN1* (10%), *RNF213* (10%), and *SSPO* (10%) (Figure 1); these alterations had not been previously reported. Previously reported alterations and indels of *CTNNA1*, *BRCA2*, *STK11*, *PRSS1*, *ATM*, *MSR1*, *PALB2*, *BRCA1*, and *RAD51C* were observed at low frequencies (median, 4 [range, 1-12] cases). As the most frequently altered gene, private germline variants of *MUC4* were observed in 53 of 284 patients (18.7%) with HDGC (Figure 2B). These genes were novel candidates and revealed a different spectrum of germline alterations for HDGC.

Because the functional significance of most missense variants is unknown, we focused our analysis on premature truncating variants (PTVs) of private variants; these PTVs included frameshift indels, nonsense alterations, and splice-site alterations according to findings of a previous study.^18^ In total, 2715 private heterozygous PTVs in 2278 genes were identified. The most altered gene, *MUC4*, had 7 PTVs, whereas *WDR87*, *CRIPAK*, *DNAH7*, *EFCAB13*, and *ITGAV* each had 4 PTVs (eFigure 2 in Supplement 1). Previous studies^30,31^ proposed that *MUC4* may induce oncogenic transformation of fibroblast cells in mice^30^ and that overexpression of *MUC4* might promote aggressive properties via *ERBB2* (formerly *HER2* or *HER2/neu*) signaling in gastric adenocarcinoma.^31^ Alterations of *ITGAV*, which is the regulator of PI3K signaling, were also previously observed in GC tumor tissues.^32^ Thus, although the functional consequences for most of the private PTVs were unknown, part of the identified private PTVs have been found to be involved in the development of GC, and these private PTVs might be associated with susceptibility to HDGC and serve as founder alterations.

### Somatic Variant Analysis

**Figure 3.**
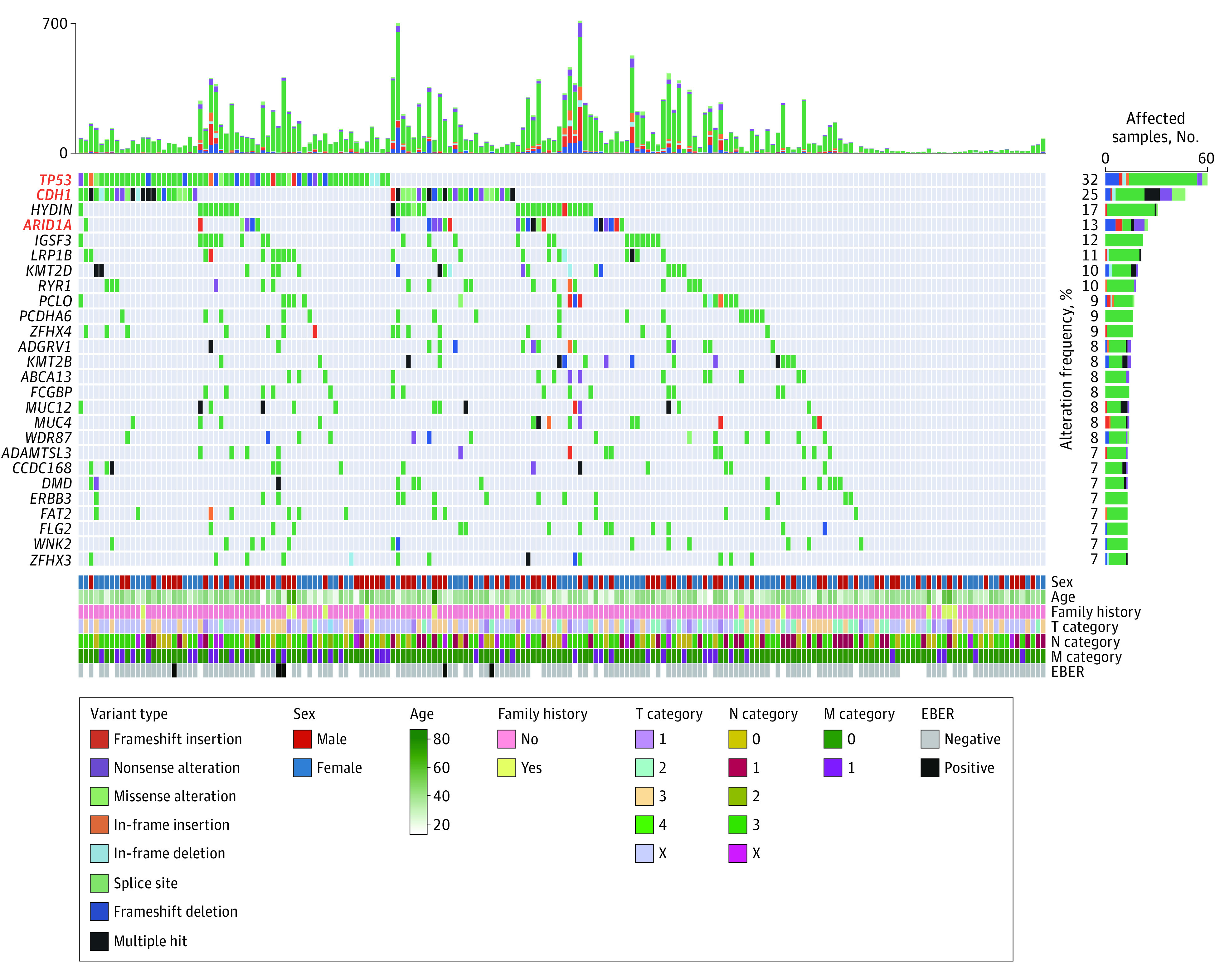
Somatic Alteration Landscape of Hereditary Diffuse Gastric Cancer Oncoplot showing the somatic alteration landscape among 186 Chinese patients with hereditary diffuse gastric cancer. The top panel shows the total of all nonsilent somatic alterations for each sample. The middle panel shows the alteration details; only the 24 most altered genes with alteration frequencies of 7% or greater are shown, with red font indicating genes with significant alterations. The bottom panel shows clinical information. Cancer staging was based on the *AJCC Cancer Staging Manual, 7th edition*. EBER indicates Epstein-Barr virus–encoded small RNAs.

**Figure 4.**
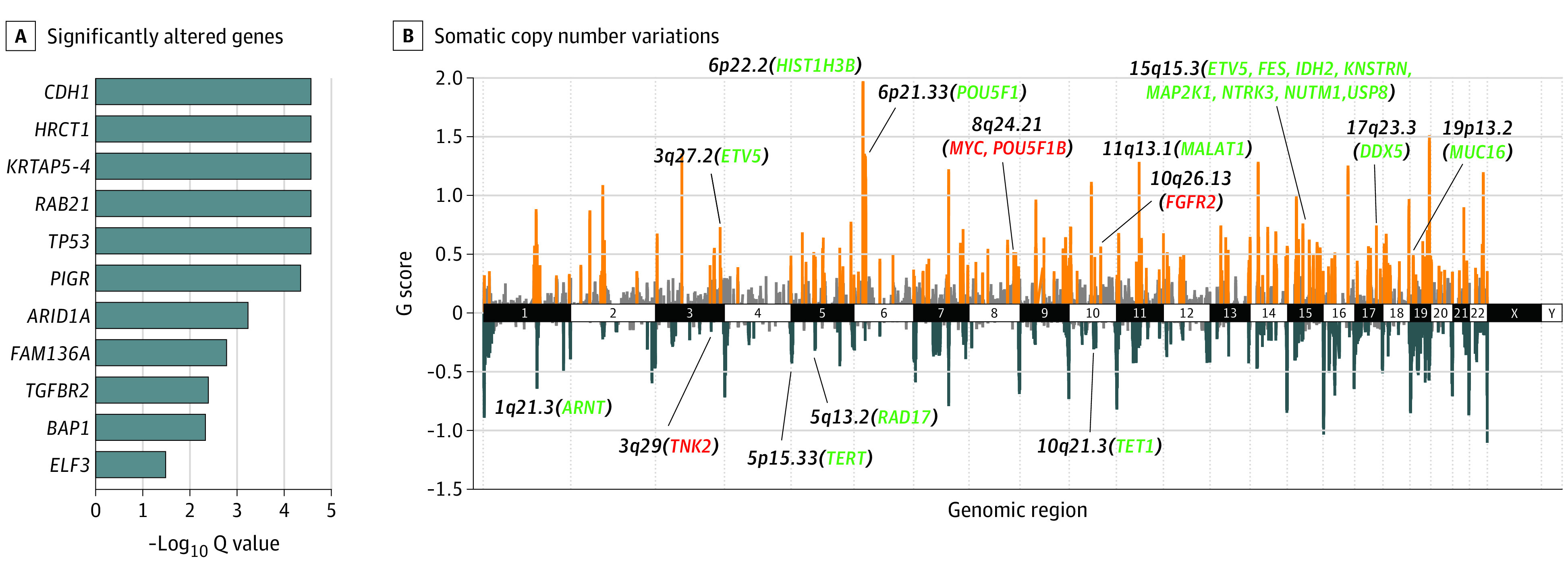
Significantly Altered Genes and Somatic Copy Number Variations A, Significantly altered genes (*Q *< .05) were detected using the MutSigCV algorithm. The low *Q* values were limited to a fixed value because of the processing precision limit. B, The G scores of genomic regions were plotted along the genome, with orange spikes representing significant copy number gains and dark gray spikes representing significant copy number losses. Light gray spikes indicate insignificant gain and loss. Key genes associated with gastric cancer are shown in red type, and cancer census genes (from the Catalogue of Somatic Mutations in Cancer signature database) associated with other tumor types are shown in green type.

In addition to germline alterations, we profiled the somatic variants for the 186 paired HDGC samples (variant validation is shown in eTable 5 in Supplement 2). The most altered gene was *TP53*, with a frequency of 32.3% (60 of 186 samples) (Figure 3). Of note, although the frequency of germline variants was low in our data set, the frequency of *CDH1* somatic alterations was as high as 25.3% (47 of 186 samples). Because only 5 *CDH1* somatic alterations were found in paired peripheral blood, with only 1 supporting read for each, the potential for constitutional mosaicism, which is a well-known mechanism for multiple hereditary cancer–associated genes, was not supported in our data set. In addition to genes with significant alterations and indels, including *TP53* (*Q *< 2.2 × 10^16^), *CDH1* (*Q *< 2.2 × 10^16^), and *ARID1A* (*Q* = 8.7 × 10^8^; 29 of 186 samples [15.6%] with alterations) (eFigure 4 in Supplement 1), which were previously identified in a TCGA cohort of patients with stomach adenocarcinoma (STAD) (11% frequency),^12^ the MutSigCV algorithm identified several *SMG* genes, including *HRCT1*, *KRTAP5-4*, *RAB21*, *PIGR*, *FAM136A*, *TGFBR2*, *BAP1*, and *ELF3*, based on our data set (Figure 4A; eTable 1 in Supplement 2).

Furthermore, we compared the alteration burden between HDGC and sporadic diffuse STAD (D-STAD) in the TCGA and found that the alteration burden of HDGC was comparable with that of D-STAD (eFigure 3A in Supplement 1); HDGC and D-STAD had similar gene alteration frequencies (*R* = 0.24; *P* < 2.2 × 10^16^) (eFigure 3B in Supplement 1). We found several highly altered genes in D-STAD that had significantly lower alteration frequencies in HDGC (eFigure 3B-C in Supplement 1). For example, the alteration frequency of *FAT4* was 22.9% in D-STAD but only 4.3% in HDGC (eFigure 3B in Supplement 1). In addition, the alteration frequency of *MLLT4* was 10.0% in D-STAD but 0% in HDGC (eFigure 3C in Supplement 1). Although HDGC had copy number variation frequencies that were relatively similar to those of D-STAD in different genomic regions (*R* = 0.37; *P* < 2.2 × 10^16^) (eFigure 3D in Supplement 1), copy number variation frequencies in many parts were significantly different, including several differences in chromosome arm-level regions (eg, more deletions of chr16p, chr17, chr19, chr20, and chr22) and many focal regions (eg, more amplification frequency in chr6p21.32-33 and chr6p22.1-2 and more deletion frequency in chr7p11, the starts of chromosomes 1 and 7, and the ends of chromosomes 8, 9, 10, and 12) (eFigure 3E in Supplement 1).

With the help of the sequencing coverage information from whole-exome sequencing, the SCNAs in HDGC were also evaluated. The chromosome-level SCNA (Figure 4B) calculated by the GISTIC2.0 algorithm suggested that a large number of SCNA events occurred in HDGC tumor tissues. Genome regions such as 6p22.2 and 19p13.2 were recurrently amplified, whereas regions such as 1q21.3 and 10q21.3 were recurrently deleted. Results of further gene-level analysis revealed that the SCNAs of known cancer-related genes, such as *PTK6* (44.6%), *ERBB3* (13.4%), *PIK3CA* (11.8%), and *UBR5* (11.8%), were relatively frequent (eFigure 4 in Supplement 1). A previous study identified the metastasis-promoting roles of *PTK6*,^33^ and Jha et al^34^ proposed that *PTK6* was under positive selection and correlated with *Helicobacter pylori* invasion; the finding that both amplification and deletion of *PTK6* were observed in the tumor was controversial (eFigure 4 in Supplement 1). Well-known oncogenes, *ERBB3* and *PIK3CA*, were reported to be involved in GC,^35,36^ and highly frequent amplification of these genes was observed in the cohort (eFigure 4 in Supplement 1). As an E3 ligase that regulates protein ubiquitination and degradation, *UBR5* has been reported to destabilize the tumor suppressor *GKN* in GC,^37^ and the frequent amplification of *UBR5* found in the present study further suggested its potential oncogenic role (eFigure 4 in Supplement 1). Although the Control-FREEC and GISTIC2.0 algorithms have been widely used and reported to be reliable,^38,39,40^ additional methods and further studies are needed to validate the SCNA results and reveal the mechanisms.

Signaling pathways, including PI3K-Akt, MAPK, cell cycle, Wnt, and TGF-β, were previously identified as important in the carcinogenesis and development of GC.^41^ In this study, we identified the somatic variant frequencies of these pathways for both the HDGC cohort (eFigure 5 in Supplement 1) and the TCGA STAD cohort (eFigure 6 in Supplement 1), which revealed distinct profiles. Generally, the somatic variant frequencies of these pathways in the TCGA cohort were substantially higher than those of the HDGC cohort. For example, the alteration rates for *PIK3CA*, *KRAS*, *SMAD4*, *MYC*, and *APC* in the TCGA STAD cohort were higher than alteration rates in the HDGC cohort (eFigure 5A-C and eFigure 6A-C in Supplement 1). The alteration rates in the cell cycle pathway were both substantially lower in the TCGA STAD cohort than the HDGC cohort (eFigure 5D and eFigure 6D in Supplement 1). Furthermore, the frequencies of SCNAs in the HDGC cohort were higher than those in the TCGA STAD cohort. Taken together, the pathways important for sporadic GC might have a smaller role in the carcinogenesis of HDGC.

### Etiologic Analysis

To investigate the etiology of HDGC, variant signature analysis was performed and correlated with the Catalogue of Somatic Mutations in Cancer (COSMIC) signature database (eMethods in Supplement 1). The results revealed that signature 1 (etiology: spontaneous deamination of 5-methylcytosine), signature 5 (etiology: unknown), and signature 24 (etiology: exposure to aflatoxin) were enriched in the cohort (eFigure 7A-B in Supplement 1), presenting a different alteration spectrum from the TCGA STAD cohort. Furthermore, the enrichment of signature 24 was consistent when the number of clusters was 4 (eFigure 7C in Supplement 1) or 5 (eFigure 7D in Supplement 1). The distribution of the 3 variant signatures among samples is presented in eFigure 7E in Supplement 1. Signature 24 was not detected in the TCGA STAD cohort but was found as a dominant signature in 11 of 186 samples (5.9%) from patients with HDGC in our study (eFigure 7F-G in Supplement 1). Patients with signature 24 predominance were enriched in the Epstein-Barr virus–positive subgroup (2 of 3 patients vs 8 of 69 patients in the virus-negative subgroup; Fisher *P* = .04) (eFigure 7H in Supplement 1). Furthermore, signature 24 was important for alterations in genes, including *TP53*, *WDR87*, *PCDH9*, *SP8*, *CNTN4*, *PIK3CA*, and *TMEM132C* (eFigure 7I in the Supplement), which might be associated with exposure to aflatoxin. These results suggested that environmental factors may have a large role in HDGC, and there might be substantial interaction between genetics and the environment.

To further elucidate the tumorigenesis mechanism, we analyzed double-hit events in HDGC. A total of 406 potential double-hit events were identified in 396 genes from 66 patients (mean, 2 [range, 0-46] events) (eTable 2 in Supplement 2). The detected count of double-hit events was associated with nonsilent germline variant count, loss of heterozygosity event count, and loss of heterozygosity region size (eFigure 8A-C in Supplement 1). In total, 35 of 66 cases (53.0%) had only 1 or 2 double-hit events, whereas there were more than 20 double-hit events from 5 cases. Only 1 double-hit event was observed in 386 genes, whereas there were 2 double-hit events for the other 10 genes. For example, 1 patient had the most double-hit events (46 events), among which *CACNA1D* was observed as a p.I264V alteration, and another patient had 2 double-hit events, among which *CACNA1D* was observed as a p.R2101Q alteration (eFigure 8D-G in Supplement 1). The double-hit events detected in another 14 COSMIC cancer census genes from patients with HDGC are shown in eFigure 9 in Supplement 1. These genes had only 1 double-hit event, suggesting insufficient evidence to support their potential roles as tumorigenesis mechanisms. Although further studies are needed to clarify the functions and mechanisms of the double-hit events, these results suggested that double-hit events might be important mechanisms in HDGC.

### Clinical Outcome Alterations and Actionable Variants

Beyond oncogenesis and cancer development, we examined clinical outcome–related variants in HDGC. The results revealed that nonsilent germline alterations in 7 genes, including SD*K1*, *HSPG2*, *FSIP2*, *CUBN*, *NCKAP5*, *FLNB*, and *MUC16*, were significantly associated with overall survival (OS) (eFigure 10A in Supplement 1). Among these genes, *MUC16* (eFigure 10B in Supplement 1) is a known oncogene annotated in the COSMIC database, and multivariate Cox proportional hazzards regression analysis revealed that the germline alterations of *NCKAP5* (eFigure 10C in Supplement 1), *HSPG2* (eFigure 10D in Supplement 1), and *FSIP2* (eFigure 10E in Supplement 1) were independent factors associated with clinical outcomes of patients with HDGC (eFigure 10F-H in Supplement 1). Furthermore, somatic alterations (including nonsilent alterations and SCNAs) in a large number of genes were found to be associated with OS (eFigure 11A in Supplement 1). Among them, COSMIC cancer census genes, including *FGFR3* (hazard ratio [HR], 2.2; 95% CI, 1.2-4.2), *ASPSCR1* (HR, 2.2; 95% CI, 1.2-4.1), *CIC* (HR, 2.4; 95% CI, 1.2-4.7), *DGCR8* (HR, 2.2; 95% CI, 1.1-4.5), and *LZTR1* (HR, 2.5; 95% CI, 1.2-5.3), were altered in high frequencies (>10%), and their alterations were independently associated with worse OS (eFigure 11B-F in Supplement 1). Because *FGFR3* is a well-known cancer-related gene, we further compared its alteration profile between the HDGC and TCGA STAD cohorts. We found that the alteration frequencies, especially the deletion frequencies, were substantially higher among those in the HDGC cohort (25.8%) vs the TCGA STAD cohort (1.4%) (eFigure 11G in Supplement 1).

Furthermore, pathway analyses found that the genes with somatic alterations that were associated with poor OS enriched interferon-related pathways, including interferon signaling, interferon α and β signaling, regulation of interferon α signaling, *DDX58-* and *IFIH1*-mediated induction of interferon α and β, antiviral response–related pathway of *TRAF6*-mediated *IRF7* activation, and factors involved in megakaryocyte development and platelet production (eFigure 11H in Supplement 1). The genes with alterations that were associated with better OS enriched tyrosine kinase–regulated pathways, including signaling by nonreceptor tyrosine kinases, signaling by *PTK6*, downregulation of *ERBB2* and *ERBB3* signaling, *ERBB2*-activating *PTK6* signaling, the canonical retinoid cycle in rods (twilight vision), and IKK complex recruitment mediated by *RIP1* (eFigure 11I in Supplement 1). These results suggest that aberrant interferon signaling and *PTK6* signaling might play important roles in the progression of HDGC.

In addition to associations with OS, the potential clinical actionability of the somatic variants was assessed according to the ESMO Scale of Clinical Actionability for Molecular Targets by integrating data from the OncoKB database,^42^ the Cancer Genome Interpreter,^43^ and the CIViC (Clinical Interpretation of Variants in Cancer) database.^44^ In total, 263 of 21 345 SSNVs (1.2%) from 125 patients with HDGC and 338 of 469 582 SCNAs (0.07%) from 153 patients with HDGC were annotated as actionable (eTable 6 in Supplement 2). The annotation results are shown in eTable 3 in Supplement 2 and eFigure 12A-B in Supplement 1. These findings suggested that actionable variants were rare for both SSNVs and SCNAs, and the distributions of evidence levels were different. According to the DrugCVar system,^45^ the evidence levels for most of the actionable SSNVs (203 of 263 [77.2%]) were higher than tier 4, whereas most of the actionable SCNAs (239 of 338 [70.7%]) were tier 4. These results might be helpful for the further development of therapies for patients with HDGC.

## Discussion

In this cohort study, we retrospectively screened more than 10 000 patients with GC over the last 16 years and established a cohort of 284 patients with HDGC, finding that 542 of 10 431 patients (5.2%) with GC met the criteria for HDGC, which was consistent with a previous estimation.^3^ Most patients in the HDGC cohort (89.4%) had no family history of diffuse GC, and they were included according to the criterion of a diffuse GC diagnosis before age 40 years. Although a previous study found that young patients, especially those younger than 35 years, had a poor prognosis,^46^ the present study found a higher 5-year OS rate in patients with HDGC (61.4%) than in patients with sporadic diffuse GC (44.1%), which suggested different biological behaviors between HDGC and sporadic diffuse GC.

In this study, the profiled genetic landscape for the HDGC cohort challenged previous observations that *CDH1* germline alteration is present in 25% to 50% of families with HDGC^28^; those incidence rates were not applicable to Chinese patients with HDGC in the current study. Even in patients with a family history of diffuse GC in this HDGC cohort, the frequency of *CDH1* germline alterations was still low (3.3%). It was previously reported that *CDH1* alterations were significantly associated with poor OS,^47^ which was consistent with the findings of our study. In addition, no highly common germline alteration was observed in this cohort, which suggested that the genetic factors associated with HDGC need to be further investigated.

In addition to the gene alterations potentially associated with carcinogenesis, we identified OS-associated gene alterations in HDGC, several of which were previously reported. For example, Li et al^48^ reported an association between *MUC16* alteration and outcomes in patients with GC, another study^12^ found that the *CIC* gene was highly altered among patients in a TCGA STAD cohort, and Yang et al^49^ found that the alteration frequencies of *MUC16* and *FSIP2* were high in East Asian patients with GC. Furthermore, we assessed the potential clinical actionability of the somatic variants. Based on analysis of SCNAs, drugs targeting amplification of mTOR, CDK4 and CDK6, fibroblast growth factor receptor, *MET,* and *ERBB2* were most common. Somatic single-nucleotide variants as drug candidates included variants of *ARID1A, PI3KCA, SMARCA4, EGFR*, and *BAP*. Until now, only drugs targeting *ERBB2* and *VEGF* have been approved for the treatment of patients with metastatic GC, and the results have been unsatisfactory. The present study provides information that could be helpful for future clinical trials of drugs targeting gene alterations in patients with HDGC.

### Limitations

This study has several limitations. First, the study is retrospective and included Chinese patients from only 2 sites. There are likely to be differences in the genetic backgrounds of East Asian vs Western populations. Further international multicenter prospective studies will be helpful to better understand the genomic features of HDGC. Second, 94.0% of patients in this study were younger than 40 years and did not have a family history of diffuse GC, which may explain the low alteration rate of *CDH1*. Additional studies including patients with different conditions, especially those with a family history or personal history of lobular breast cancer, are warranted because patients with different conditions were not included in the present cohort. Third, the incidence rate of actionable altered genes was low, which may limit the application of this study’s findings, and the diverse chemotherapy treatments received by patients with HDGC in this study made it difficult to control for factors to assess chemotherapy prognosis-related events, such as homologous recombination repair gene alterations.

## Conclusions

This cohort study provides a genetic landscape for HDGC that challenges the previously reported high germline alteration rate of *CDH1* in HDGC and identified new potential susceptibility genes. Analyses of variant signatures and double-hit events revealed important mechanisms for HDGC tumorigenesis. To our knowledge, the present study is the first to provide such a genetic picture for HDGC, which may be helpful for the development of diagnostic assessments and therapies for patients with GC.

## Notes

Supplement 1. **eMethods.** Patient Enrollment, Whole-Exome Sequencing of HDGC Samples, Target Capture Sequencing, SNV/INDEL and Somatic Copy Number Alterations, Double-Hit Event Analysis of HDGC, Drug Target Analysis, and Statistical Analyses **eResults.** HDGC Cohort and Exome Sequencing **eFigure 1.** Flowchart Illustrating Filter-Based Strategy to Identify Novel Candidate HDGC Susceptibility Genes at Both Germline and Somatic Levels **eFigure 2.** Truncating Germline Alteration Landscape of HDGC **eFigure 3.** Comparison Between HDGC and TCGA Data **eFigure 4.** Oncoplot Illustrating Somatic Alterations and Copy Number Alterations of the COSMIC Cancer Gene Census **eFigure 5.** Variant Summary Diagram of Gastric Cancer–Related Pathways in HDGC **eFigure 6.** Variant Summary Diagram of Gastric Cancer–Related Pathways in TCGA Stomach Cancer Cohort **eFigure 7.** Somatic Alteration Signatures of HDGC **eFigure 8.** Correlation Analyses and Examples of Double-Hit Events **eFigure 9.** Double-Hit Events Detected in COSMIC Cancer Census Genes From Patients With HDGC **eFigure 10.** Clinical-Associated Variants in HDGC **eFigure 11.** Clinical-Associated Somatic Alterations (Nonsilent Alterations and CNAs) in HDGC **eFigure 12.** Summary of Actionable Variants of HDGC
eReferences

Supplement 2. **eTable 1.** Results of MutSigCV Analysis of Somatic Alterations **eTable 2.** Double-Hit Events in HDGC **eTable 3.** Actionable Somatic SNVs in HDGC Cohort **eTable 4.** Complete List of Whole-Exome Sequencing Data With Detailed Information **eTable 5.** Variant Validation Results by Target Sequencing **eTable 6.** Mapping Details Among ESCAT, OncoKB, CIViC, and CGI

Supplement 3.
Data Sharing Statement
