# Supplementary material for: Whole-Exome Sequencing Among Chinese Patients With Hereditary Diffuse Gastric Cancer
Source: JAMA Netw Open. 2022 Dec 9;5(12):e2245836. doi: 10.1001/jamanetworkopen.2022.45836 (PMC9856492; doi:10.1001/jamanetworkopen.2022.45836)
Supplement: Supplement 1. — eMethods. Patient Enrollment, Whole-Exome Sequencing of HDGC Samples, Target Capture Sequencing, SNV/INDEL and Somatic Copy Number Alterations, Double-Hit Event Analysis of HDGC, Drug Target Analysis, and Statistical Analyses eResults. HDGC Cohort and Exome Sequencing eFigure 1. Flowchart Illustrating Filter-Based Strategy to Identify Novel Candidate HDGC Susceptibility Genes at Both Germline and Somatic Levels eFigure 2. Truncating Germline Alteration Landscape of HDGC eFigure 3. Comparison Between HDGC and TCGA Data eFigure 4. Oncoplot Illustrating Somatic Alterations and Copy Number Alterations of the COSMIC Cancer Gene Census eFigure 5. Variant Summary Diagram of Gastric Cancer–Related Pathways in HDGC eFigure 6. Variant Summary Diagram of Gastric Cancer–Related Pathways in TCGA Stomach Cancer Cohort eFigure 7. Somatic Alteration Signatures of HDGC eFigure 8. Correlation Analyses and Examples of Double-Hit Events eFigure 9. Double-Hit Events Detected in COSMIC Cancer Census Genes From Patients With HDGC eFigure 10. Clinical-Associated Variants in HDGC eFigure 11. Clinical-Associated Somatic Alterations (Nonsilent Alterations and CNAs) in HDGC eFigure 12. Summary of Actionable Variants of HDGC eReferences [file jamanetwopen-e2245836-s001.pdf]

Supplemental Online Content

Liu ZX, Zhang XL, Zhao Q, et al. Whole-exome sequencing among Chinese patients with hereditary diffuse gastric cancer. *JAMA Netw Open*. 2022;5(12):e2245836. doi:10.1001/jamanetworkopen.2022.45836

eMethods. Patient Enrollment, Whole-Exome Sequencing of HDGC Samples, Target Capture Sequencing, SNV/INDEL and Somatic Copy Number Alterations, Double-Hit Event Analysis of HDGC, Drug Target Analysis, and Statistical Analyses

eResults. HDGC Cohort and Exome Sequencing

eFigure 1. Flowchart Illustrating Filter-Based Strategy to Identify Novel Candidate HDGC Susceptibility Genes at Both Germline and Somatic Levels

eFigure 2. Truncating Germline Alteration Landscape of HDGC

eFigure 3. Comparison Between HDGC and TCGA Data

eFigure 4. Oncoplot Illustrating Somatic Alterations and Copy Number Alterations of the COSMIC Cancer Gene Census

eFigure 5. Variant Summary Diagram of Gastric Cancer–Related Pathways in HDGC

eFigure 6. Variant Summary Diagram of Gastric Cancer–Related Pathways in TCGA Stomach Cancer Cohort

eFigure 7. Somatic Alteration Signatures of HDGC

eFigure 8. Correlation Analyses and Examples of Double-Hit Events

eFigure 9. Double-Hit Events Detected in COSMIC Cancer Census Genes From Patients With HDGC

eFigure 10. Clinical-Associated Variants in HDGC

eFigure 11. Clinical-Associated Somatic Alterations (Nonsilent Alterations and CNAs) in HDGC

eFigure 12. Summary of Actionable Variants of HDGC

eReferences

This supplemental material has been provided by the authors to give readers additional information about their work.

© 2022 Liu ZX et al. *JAMA Network Open*.

eMethods. Patient Enrollment, Whole-Exome Sequencing of HDGC Samples, Target Capture Sequencing, SNV/INDEL and Somatic Copy Number Alterations, Double-Hit Event Analysis of HDGC, Drug Target Analysis, and Statistical Analyses

Hereditary diffuse gastric cancer (HDGC) sample enrollment

Patients with HDGC met at least one of the following four criteria [1]:

i) Two or more cases of GC, one confirmed case of diffuse GC in someone younger than 50 years; ii) Three or more confirmed diffuse GC cases in the first- or second-degree relatives, independent of age of onset; iii) Diffuse GC before the age 40 and without a family history; iv) Personal or family history of diffuse GC and lobular breast cancer, one of which must have been diagnosed before the age 50. All HDGC samples were enrolled from Sun Yat-sen University Cancer Center (N=282) and The First People's Hospital of Foshan (N=2). All cancer diagnoses in each kindred were pathologically confirmed. The clinicopathological characteristics of enrolled patients were summarized in Table 1.

Whole-exome sequencing (WES) of HDGC samples

As shown in Figure S1, we screened the clinical data of 10,431 GC patients who were diagnosed at Sun Yat-sen University Cancer Center (SYSUCC) between January 2002 and August 2018. There were 542 patients who met the criteria of HDGC, patients with insufficient leukocyte samples (n=177) or without detailed follow-up (n=83) were excluded. Another 2 patients from The First People's Hospital of Foshan nearby SYSUCC were also included, and they were from the same family with 5 diffuse GC patients. Finally, this retrospective HDGC cohort included 284 HDGC patients and we analyzed the data in August 2020. A total of 284 HDGC leukocyte samples and 186 paired tumor samples were subjected to WES. Briefly, a

© 2022 Liu ZX et al. *JAMA Network Open*.

total of 0.8 μg genomic DNA per sample with high molecular weight (> 20 Kb single band) was used for DNA library preparation. The sequencing libraries were constructed according to the manufacturer’s recommendations using a TruSeq Nano DNA HT Sample Prep Kit (Illumina, USA), and index codes were added to each sample. Exonic regions were captured in solution using the Agilent SureSelect v.6 kit (Agilent Technologies) according to the manufacturer's instructions. Then, the DNA libraries were sequenced using the Illumina Whole Genome Sequencing Service with the HiSeq X platform, generating 2 × 150 bp reads per fragment in the final library (Table S4).

Target Capture Sequencing

The genomic DNA (gDNA, 1ug) was fragmented for a target peak of 180 to 250bp using Covaris Series sonicator, and then submitted for size selection by purification beads. Blunt-end DNA fragments were generated by a combination of fill-in reaction and exonuclease activity. An A-base was then added to the blunt ends of each strand, preparing them for ligation to the T-tail sequencing adapters. 6 cycles of Polymerase Chain Reaction (PCR) were performed for amplification of the ligation products to generate the gDNA library for target capturing. The prepared gDNA libraries were hybridized with VariantBaits Custom capture probe (0.70Mb) in 65℃ for 24h. After hybridization, the enriched libraries were captured on streptavidin beads, followed by 14 cycles of PCR amplification of capture products. Purification of amplification products was performed using purification beads to generate final capture library.

© 2022 Liu ZX et al. *JAMA Network Open*.

SNV/INDEL and somatic copy number alterations (SCNA) calling from WES

Sequence reads were aligned to the human reference genome (UCSC hg19) using the Burrows–Wheeler Aligner (BWA), allowing for two mismatches in the 30-base seed. Picard-tools were used to correct mate-pair mismatch, remove duplicate reads, and assess target region coverage. The Genome Analysis Toolkit version 3.8 (GATK3) was used following The Best Practice tutorial with default parameters to generate SNVs and small INDEL calls in the target regions, after local realignment around INDELs and base recalibration with the Unified Genotyper.

Germline SNPs and INDELs were called by HaplotypeCaller from GATK with default parameters and functionally annotated by ANNOVAR. To evaluate the most confident SNPs and INDELs related to HDGC, we performed the following criteria:

1. The allele frequency of heterozygous mutations called by HaplotypeCaller is in the range of 0.33 to 0.66;

2. Sequencing depth of mutation site is more than 10 and there are more than 5 mutant reads;

3. Reads covering one mutation site are uniquely remapped to hg38 by BLAT;

4. Mutation frequency is less than 1% in normal public database including 1000 Genome Project (1000GP, 2015 Aug, http://www.internationalgenome.org/), ESP6500 (version esp6500siv2, https://esp.gs.washington.edu/drupal/), ExAC (version ExAC03, http://exac.broadinstitute.org/) databases and BGI CHINESE MILLIONOME DATABASE

© 2022 Liu ZX et al. *JAMA Network Open*.

(CMDB);

5. Privately mutated among all patients.

The private variants were defined through the following criteria as follows: (i) nonsense variants, splice-site variants, and frameshift INDELs; (ii) heterozygous in the germline; (iii) less than 0.5% minor allele frequency (MAF) in the 1000 Genomes Project [2] or the CMDB [3]; (iv) present in only one patient; (v) more than 0.5 mappability score; and (vi) no more additional genomic locus through BLAT based query [4].

Since the most known high-penetrance disease-associated variants are located in the coding regions, only genetic variants located in these regions were considered. Moreover, as the functional impact of most missense variants is obscure, we focus on truncating variants (including nonsense mutations, splice site mutations, and frameshift INDELs), which usually result in the function loss of the encoded proteins.

Somatic SNVs and INDELs were analyzed using Mutect2 with default parameters. We also extracted reads mapping on the SNV/INDELs and remap them on human reference hg38 using BLAT, and filtered the mutations whose reads were mapped in more than one position of hg38. Considering the oxidative deamination in FFPE samples, we evaluated the effects of C>T/G>A artefacts in our data. We separated SNVs into low frequency SNVs and high frequency SNVs basing on the variant allele frequency (VAF) cut-off 0.1, and compared the fractions of C>T/G>A in both SNV groups. There is no significant fraction difference between SNVs which VAF < 0.1 (29.26%) with that of VAF > 0.1 (31.77), and there are more C>T/G>A SNVs in the high frequency group. This result indicated the FFPE-induced artifact is negligible

© 2022 Liu ZX et al. *JAMA Network Open*.

and make few influences in the data analysis. Somatic variants in variant call format (VCF) were annotated using ANNOVAR. Then MuSigCV (v1.41) was used to define significantly mutated genes (SMGs) in the somatic HDGC cohort. A gene with a q-value less than 0.05 was considered to be significantly mutated (Table S1). Mutational signature analysis was performed using maftools v 2.2.10 [5]. Three signatures were decomposed by the non-negative matrix factorization (NMF) method. Each signature was compared against known signatures derived from the COSMIC database (https://cancer.sanger.ac.uk/cosmic/signatures_v2) based on the cosine similarity. To show the frequently mutated genes in the oncoplot, we filtered the mutations frequently detected (minor allele frequency (MAF) > 0.01) in databases including 1000GP, ESP6500, ExAC and CMDB. MSI status was determined by MSIsensor (v0.5) [6] and samples with an MSI score > 10 were considered as MSI-H.

To validate the detected germline mutation as well as somatic mutations, we performed ultra-deep targeted sequencing of 100 genes including 55 most frequently germline mutated genes and 45 somatic mutated genes, which is mentioned before. Target sequencing reads were aligned to the hg19 using the BWA with the same parameters of WES, and "samtools mpileup" was performed to investigate both sequencing depth as well as mutant reads depth of each mutation site. The mutation sites with limited sequencing depth (reads count < 50) were removed, and mutation sites with mutant depth more than 3 were treated as validated sites (Table S5).

SCNAs were detected using Control-FREEC v11.1. The GISTIC2 algorithm was used to infer recurrently amplified or deleted genomic regions in the HDGC cohort. G-scores were

© 2022 Liu ZX et al. *JAMA Network Open*.

calculated for sequencing regions based on the frequency and amplitude of amplification or deletions affecting each gene. The “high-level amplification (or deletion) thresholds of segment mean” provided by GISTICS2 was used to define “amplification (AMP)” and “deletion (DEL)” at the gene level.

Double-hit event analysis of HDGC

Firstly, the purity of each tumor sample was evaluated based on the ABSOLUTE (v1.0.6) algorithm. Both SCNA segmentation files and SNV files in VCF format were set as input to ABSOLUTE. In line with the recommended best practice, all ABSOLUTE solutions were reviewed by 3 bioinformaticians, with solutions selected based on the majority vote.

The loss of heterozygosity (LOH) regions was detected using Control-FREEC v11.1, which could calculate the tumor coverage relative to germline (logR) and b-allele frequencies (BAF) of each 1000GP SNP site, and further define the LOH regions.

Double-hit event was defined as the heterozygous germline mutation which becomes homozygous because of the LOH, which may expand the impact of germline mutation and potentially relate to tumorigenesis. We selected high-quality heterozygous germline mutations located in the LOH regions and extracted the sequencing depth and mutant reads count of each selected germline mutation site in tumor samples using Samtools “mpileup” function with the parameter “-p 20 -P 20”. We calculated the expected VAF in the bulk tissue sample with specific tumor purity using the following formula:

$$VAF_{exp} = 0.5 \times (1 - Purity) + Purity$$

© 2022 Liu ZX et al. *JAMA Network Open*.

We simulated sequencing in the specific read depth of one mutation site with the expected VAF for 10^5 times and calculated the 95% confidence interval of expected VAF (VAF_{exp}) of each given germline mutation site. We identified germline mutations for which the detected VAFs in tumor sequencing data were more than 0.5 and in the 95% confidence intervals and defined them as double-hit events (Table S2).

Drug target analysis

All variants including somatic mutations and gene-level CNAs were queried for potential actionability in three drug databases including OncoKB[7], Cancer Genome Interpreter (CGI) [8] and CIVIC [9]. The common data model proposed by the ESMO Scale for Clinical Actionability of molecular Targets (ESCAT) [10] which includes 6 tiers of different actionable levels was employed. We integrated these three databases into this criterion and added the levels of “R1” and “R2” from OncoKB into the common evidence tiers, which represented different levels of drug resistance predictions. The mapping details are shown in Table S6.

Statistical analyses

For comparison between categorized variables, two-sided *P* values were calculated with the Fisher’s Exact Test or chi-square test. For multiple testing corrections, the false discovery rate was calculated using the Benjamini-Hochberg procedure. Survival function estimation was performed using the Kaplan–Meier estimates and the log-rank test. All statistical analyses were performed under R environment (version 3.5.0). Statistical significance was set at two-sided $P < 0.05$.

© 2022 Liu ZX et al. *JAMA Network Open*.

eResults. HDGC Cohort and Exome Sequencing

The clinicopathological characteristics of the final 284 HDGC patients were summarized in Table 1. Young patients were predominant in this cohort that 94% of the patients were younger than 40 years old. Since the ratio of male to female was about 2 in sporadic GC patients, a predominance of females (56.7%) was also observed. Only 2.8% (8/284) of the patients were EBV positive, which was lower than the previously reports in GC [11]. AJCC 7th TNM stage III-IV accounted for 66.9% of the cohort. Till December 31, 2018, the median follow-up was 21.7 months. The 5-year survival rate for the cohort was 61.4% (95%CI: 53.2%-68.6%), which was higher than our previous report in sporadic diffuse GC patients (44.1%) [12].

Based on the established cohort, sequencing and data analysis were carried out according to the procedures presented in Figure S1. WES was performed in peripheral white leukocyte cell DNA for all the patients, with an average depth of 215-fold (range 40-382, median 190). Fresh-frozen or Formalin-Fixed Paraffin-Embedded (FFPE) tissue specimens from 186 patients were also submitted to exome sequencing with an average depth of 274-fold (range 77-427, median 277) (Table S4). The microsatellite instability status determined by MSIsensor showed that all the samples were microsatellite stable. Since HDGC is a rare disease, the analysis of germline variants was focused on private variants, similar to a previous study on familial pancreatic cancer [13]. Based on these data and criteria for private variant, an average of 143.62 (range 0-226) and a total of 40,788 private non-silent germline single-nucleotide variants (GSNVs) were identified. Furthermore, an average of 1.44 (range 0-6, 410 in total) private small insertions and 3.04 (range 0-11, 863 in total) private small

© 2022 Liu ZX et al. *JAMA Network Open*.

deletions per patient were detected, with average lengths of 5.89 bp (range 1-100 bp) and 5.67 bp (range 1-183 bp), respectively (Figure S2). From the tumor tissue-based sequencing, an average of 122.50 non-silent somatic SNVs (SSNV) (range 1-631, 22,785 in total), 13.40 (range 0-323, 2,492 in total) insertions and 10.86 (range 0-155, 2,020 in total) deletions per patient were detected.

To validate the variants from WES data, we performed ultra-deep targeted sequencing of 100 genes, including 55 most frequently germline mutated genes and 45 somatic mutated genes. The panel-based sequencing generated the data with an average depth of 1412-fold (range 210-4100) for FFPE tissue specimens. Among the detected 320 single nucleotide polymorphisms (SNPs) and 9 INDELs for the 55 most frequently germline mutated genes, 6.56% (21/320) and 11.11% (1/9) were removed due to the limited sequencing depth (<50X), while 95.99% (287/299) and 100% (8/8) were validated as consistent with WES, respectively (Table S5). For the 117 single nucleotide variants and 48 INDELs in the 45 somatic mutated genes, 5.98% (7/117) and 14.58% (7/48) were removed due to the limited sequencing depth (<50X), while 96.36% (106/110) and 92.68% (38/41) were validated as consistent with WES, respectively (Table S5). Taken together, these results showed that the variants calling in this study was highly reliable.

© 2022 Liu ZX et al. *JAMA Network Open*.

eFigure 1. Flowchart Illustrating Filter-Based Strategy to Identify Novel Candidate HDGC Susceptibility Genes at Both Germline and Somatic Levels

See also Methods

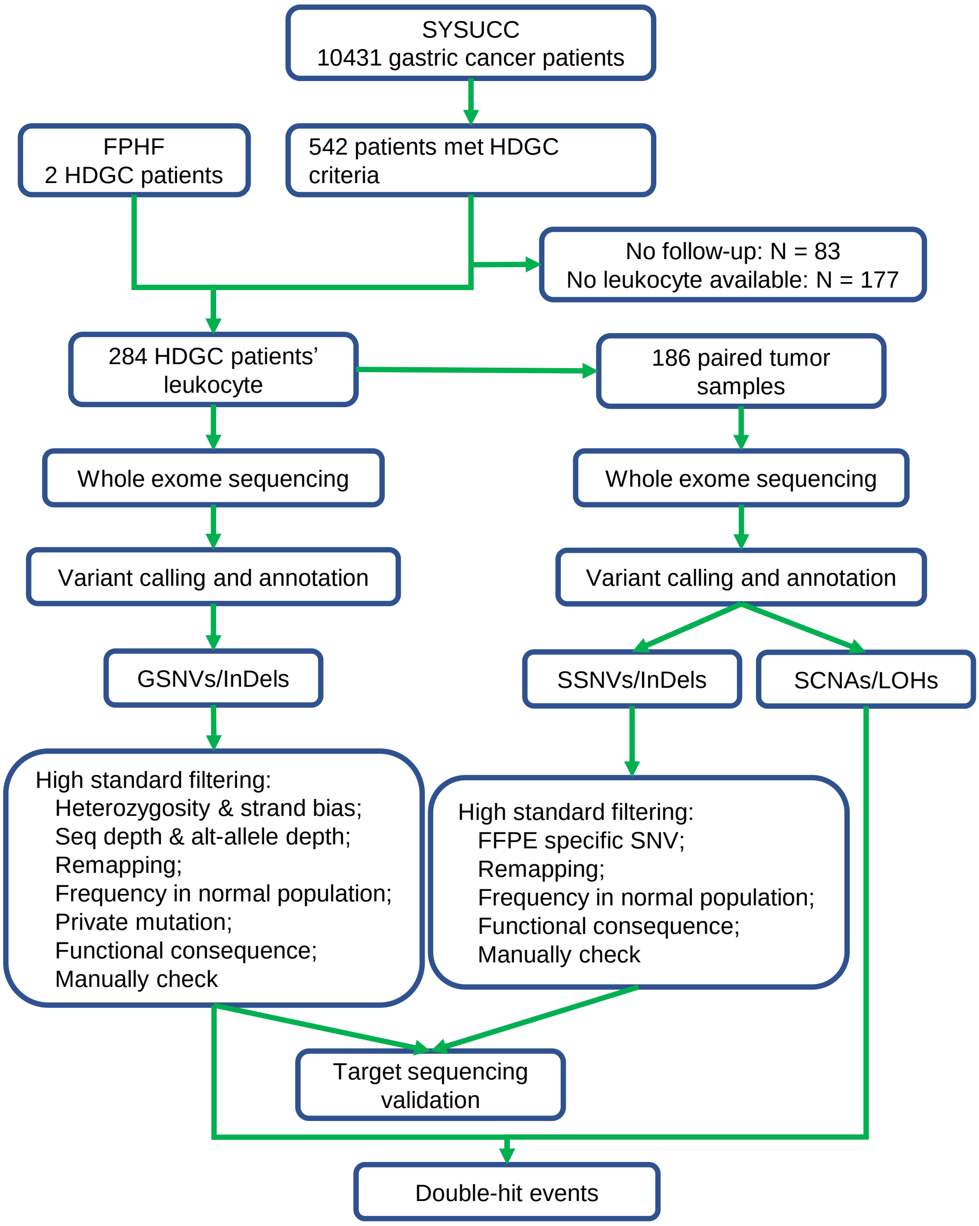

© 2022 Liu ZX et al. *JAMA Network Open*.

eFigure 2. Truncating Germline Alteration Landscape of HDGC

The top panel shows the total truncate germline mutations for each sample. The middle panel shows the mutation details (only genes with mutation frequency no less than 1% are shown). Right panel shows the number of affected samples for each gene. Clinical information is shown in the bottom panel.

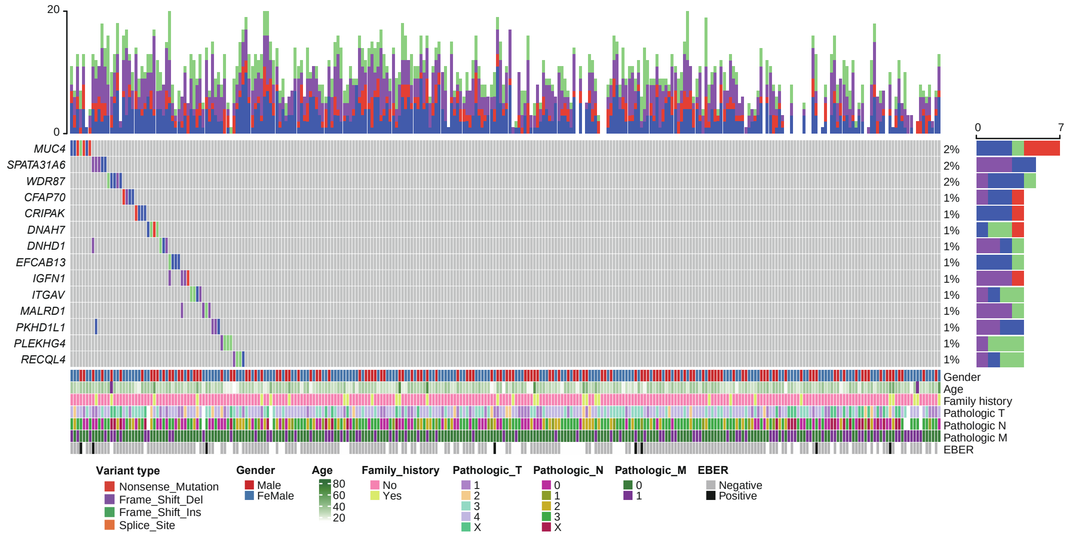

© 2022 Liu ZX et al. *JAMA Network Open*.

eFigure 3. Comparison Between HDGC and TCGA Data

A. Comparison of the number of the non-silent mutations detected in HDGC and TCGA diffused stomach adenocarcinoma (D-STAD). ns: No significance. B. Linear relationship of gene mutation frequency between DHGC and D-STAD. The red dots present the differentially mutated genes (DMGs, adjusted *P* < 0.05, Chi-square test) and the green dots present the other genes. Top genes with the most mutation frequency are marked in white and DMGs are marked in blue. The lightblue shaded area along the regression line corresponds to the 95% confidence level interval obtained from the linear model. C. Co-oncoplot shows the differentially mutated genes between HDGC cohort and TCGA gastric cancer cohort. Genes included in COSMIC cancer census are marked in red color. D. Linear relationship of CNA frequency in different chromosome regions between DHGC and D-STAD. Each dots represent a chromosome cytobands (significantly different cytobands in red and others in green). The lightblue shaded area along the regression line corresponds to the 95% confidence level interval obtained from the linear model. The significance are determined as adjusted *P* < 0.05 by Chi-square test. E. Genome-wide frequency plot of copy number alterations for HDGC (top) and D-STAD (middle) samples, respectively. All amplifications regions are shown in red, and all deletions are shown in blue. Chi-square test is performed for each cytoband, and the *P*-value distribution of each region was shown in the bottom module. The green lines stand for the adjusted P-value thresholds of significance of amplification and deletion, respectively.

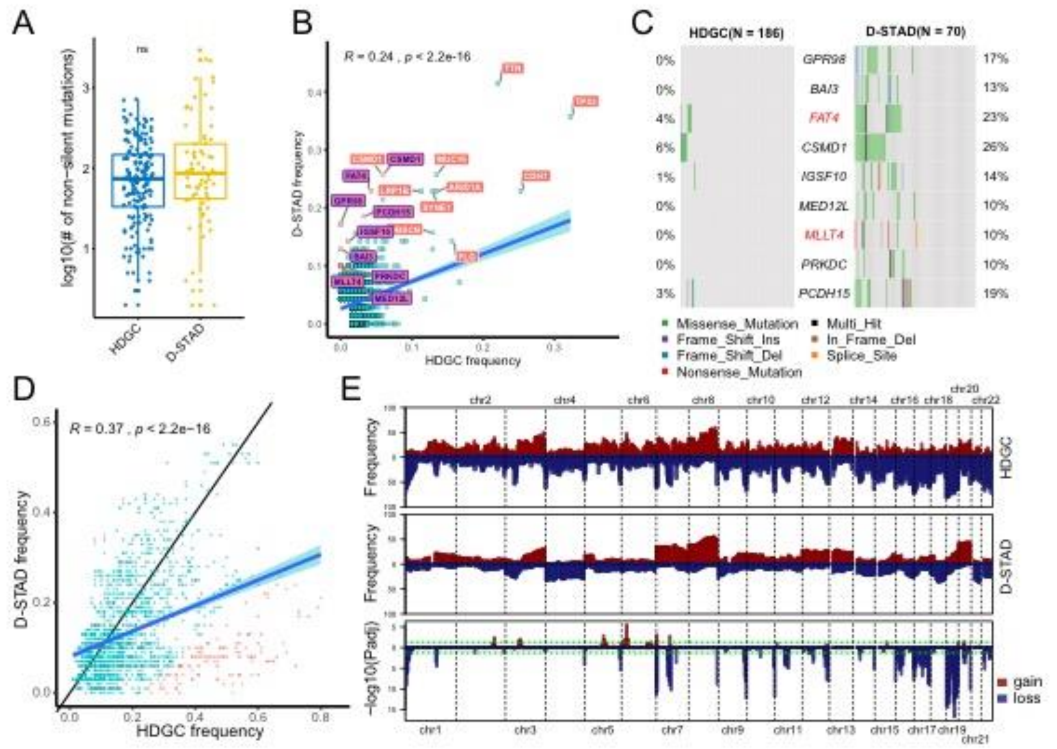

© 2022 Liu ZX et al. *JAMA Network Open*.

eFigure 4. Oncoplot Illustrating Somatic Alterations and Copy Number Alterations of the COSMIC Cancer Gene Census (CGC)

The top 22 genes (alteration frequency ≥ 4%) are shown. The total number of mutations and CNAs of CGC are shown in separated barplot in the top panel.

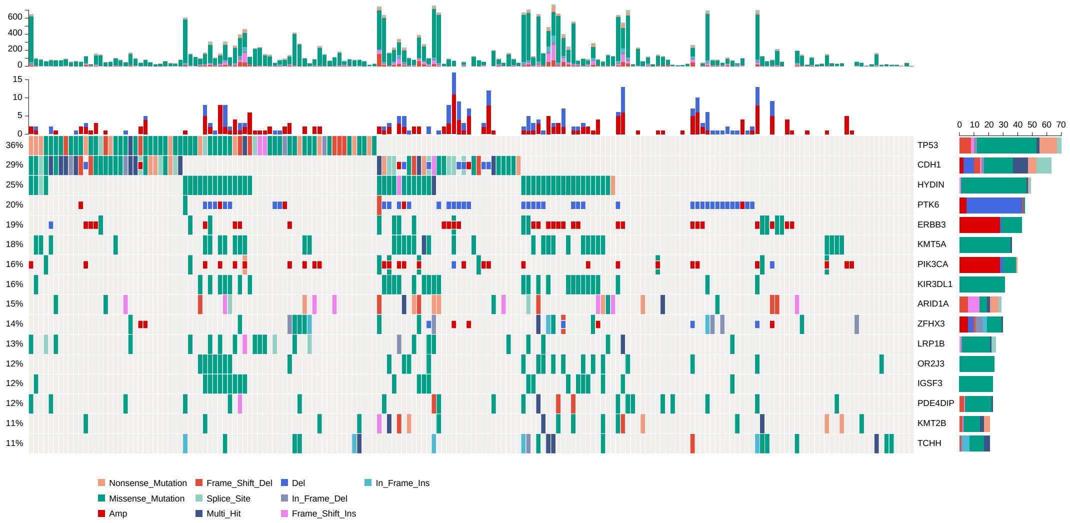

© 2022 Liu ZX et al. *JAMA Network Open*.

eFigure 5. Variant Summary Diagram of Gastric Cancer–Related Pathways in HDGC

Identified somatic mutations, germline mutations and somatic CNAs involved in important pathways of gastric cancer, including PI3K-Akt signaling pathway and MAPK pathway (A), Cell cycle (B), Pathways in cancer and Wnt signaling pathway (C) as well as TGF-β signaling pathway (D), are summarized. variant frequencies were illustrated in gradient colors.

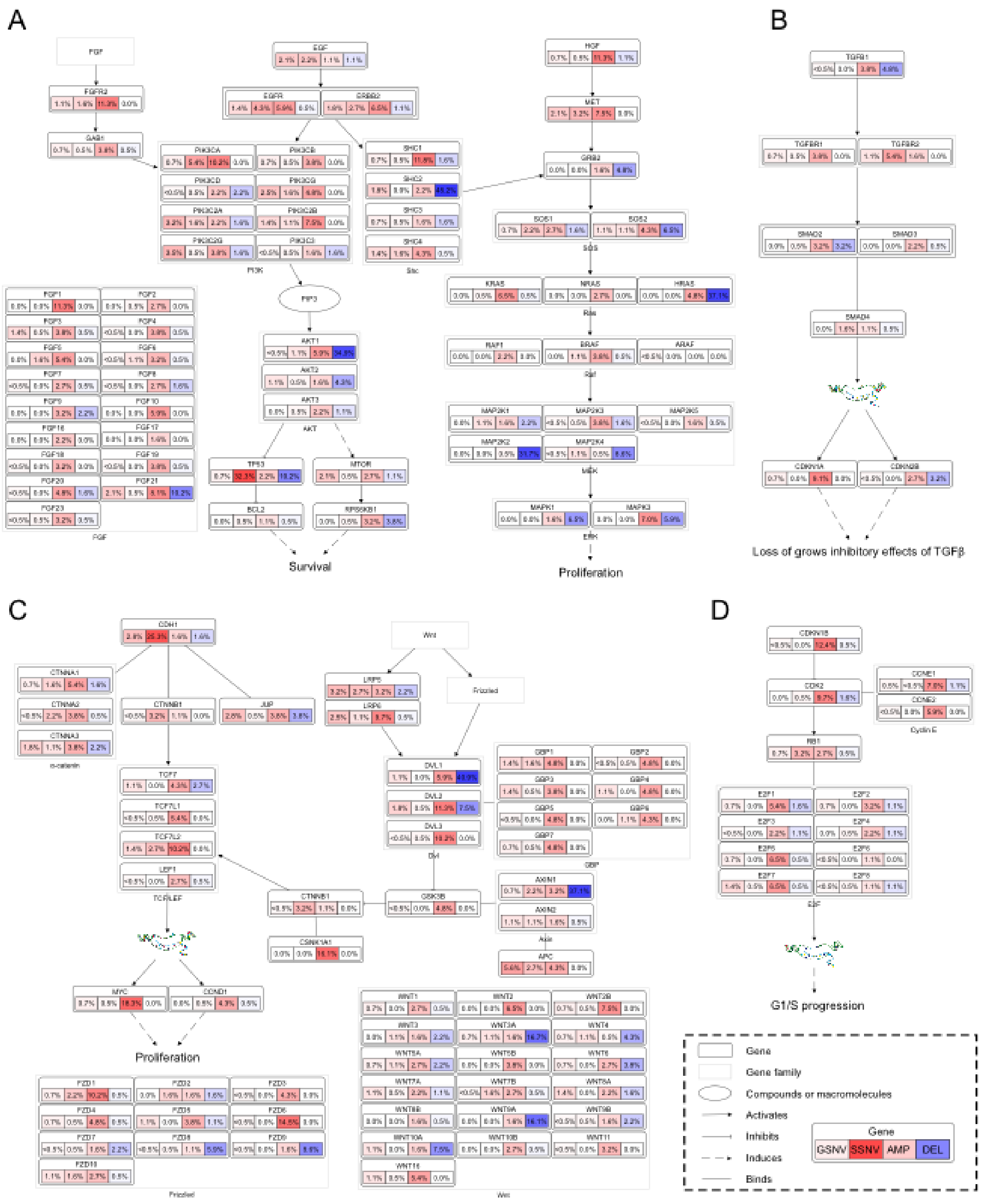

© 2022 Liu ZX et al. *JAMA Network Open*.

eFigure 6. Variant Summary Diagram of Gastric Cancer–Related Pathways in TCGA Stomach Cancer Cohort

Identified somatic mutations, germline mutations and somatic CNAs involved in important pathways of gastric cancer, including PI3K-Akt signaling pathway and MAPK pathway (A), Cell cycle (B), Pathways in cancer and Wnt signaling pathway (C) as well as TGF-β signaling pathway (D), are summarized. Variant frequency was illustrated in gradient colors.

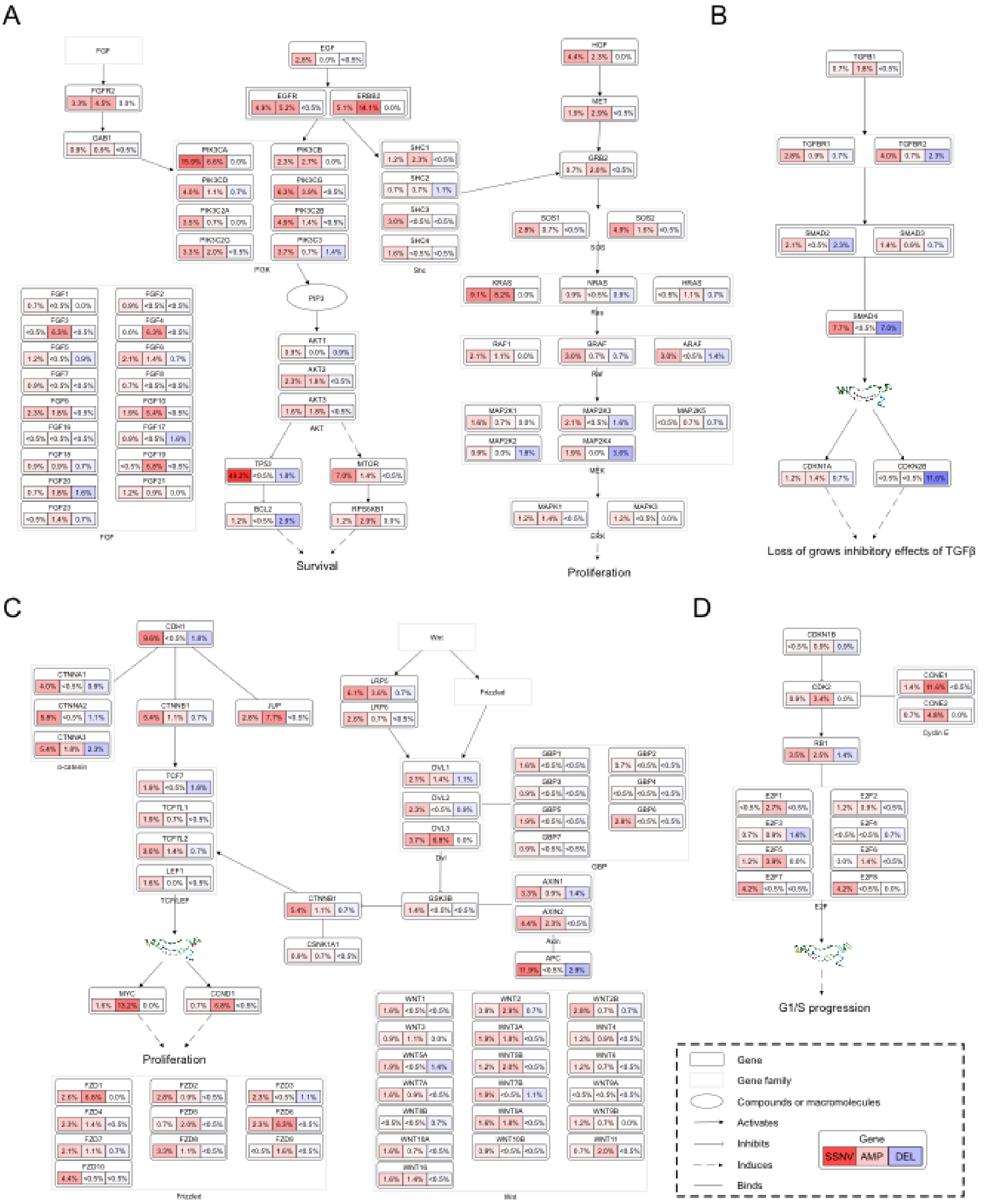

© 2022 Liu ZX et al. *JAMA Network Open*.

eFigure 7. Somatic Alteration Signatures of HDGC

A. Cophenetic correlation coefficients associated with different numbers of clusters unsupervised clustered using nonnegative matrix factorization (NMF). N=3 was selected as the best choice for mutation signature decomposition. B-D. Mutation signatures decomposed by NMF method. Each signature is compared against known signatures derived from the COSMIC database (https://cancer.sanger.ac.uk/cosmic/signatures_v2) based on the cosine similarity. E. Mutation signatures distribution along samples. Mutation signatures are decomposed by nonnegative matrix factorization (NMF) method. Each signature is compared against known signatures derived from the COSMIC database (https://cancer.sanger.ac.uk/cosmic/signatures_v2) based on the cosine similarity. F. Heatmap of mutation signature contributions of cluster centers (means of contribution) which separated using K-mean clustering algorithm basing on signature contributions in each tumor sample. We determined K = 3 so that each sample cluster could be assigned into a different mutation signature, separately. G. PCA plot shows samples assigned with different clusters mentioned in C. H. Signature cluster distribution summarized according to different clinical subgroups. I. Differentially enriched genes for COSMIC signature 24-like signature. Pairwise and groupwise fisher exact tests were performed for each gene, and genes with P-value < 0.05 were shown. Genes with mutations less than 5 are ignored in this analysis. A proportion value is calculated by the number of a specific signature (or rests) mutations in one gene divided by all mutation counts of this mutation signature (or rests).

© 2022 Liu ZX et al. *JAMA Network Open*.

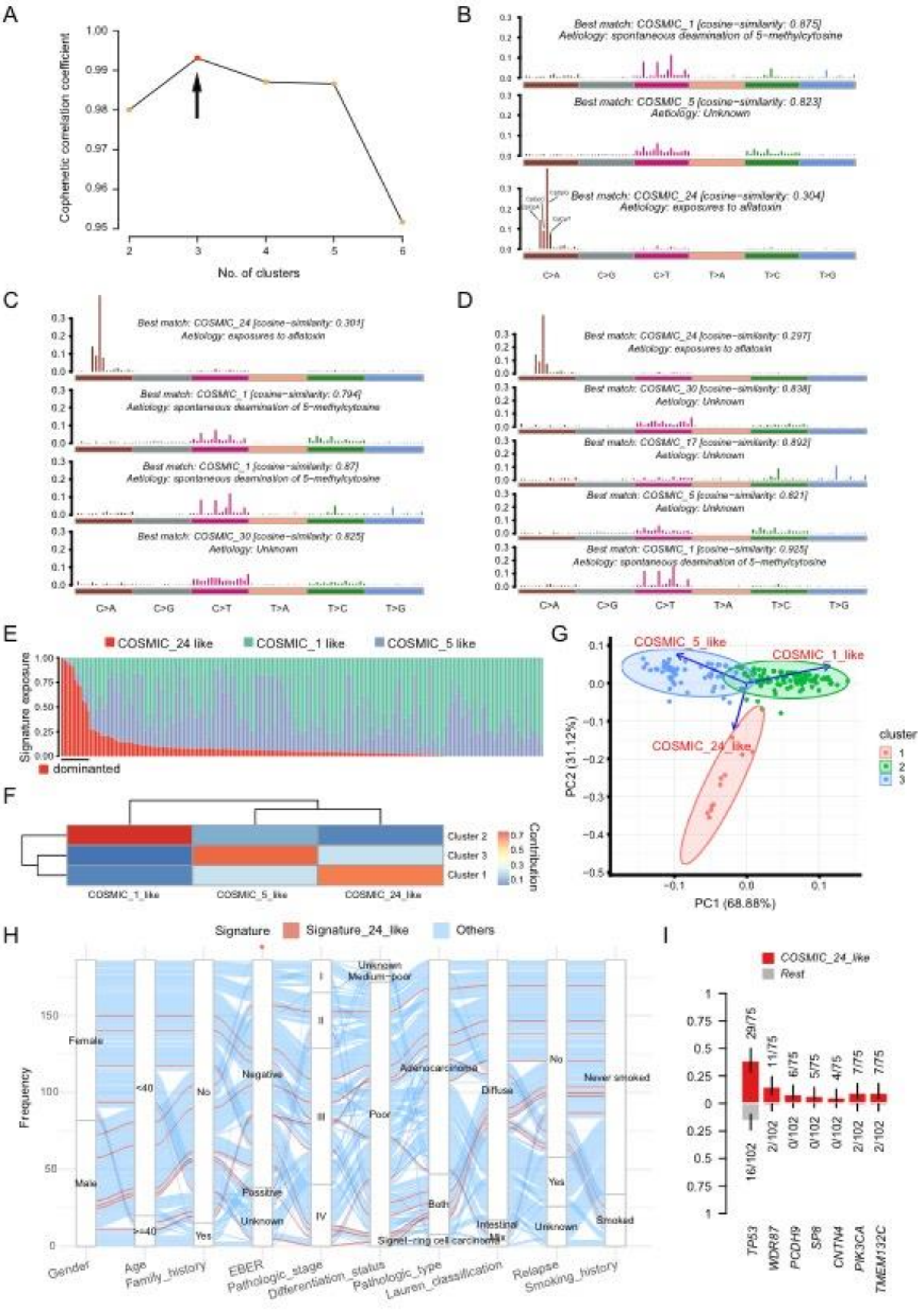

© 2022 Liu ZX et al. *JAMA Network Open*.

eFigure 8. Correlation Analyses and Examples of Double-Hit Events

The correlation between the detected count of double-hit events and germline mutation count (A), LOH event count (B), LOH region size (C). D. Reads mapping to the mutation point sites of *CNCAN1D* in patient CHGC037. E. Copy-number variant and allelic imbalances of *CNCAN1D* in patient CHGC037. F. Reads mapping to the mutation point sites of *CNCAN1D* in patient CHGC091. G. Copy-number variant and allelic imbalances of *CNCAN1D* in patient CHGC091. Forward- and Reverse-mapping reads are colored in red and blue, respectively. Copy-number variant profile is represented by log R ratio (LRR), centered at zero. Red points: amplificated regions; Blue points: deleted regions; Green points: neutral regions. Allelic imbalances are represented by B-allele frequency (BAF). Blue points: LOH regions; Red points: no-LOH regions. The germline mutation sites are marked by triangles.

© 2022 Liu ZX et al. *JAMA Network Open*.

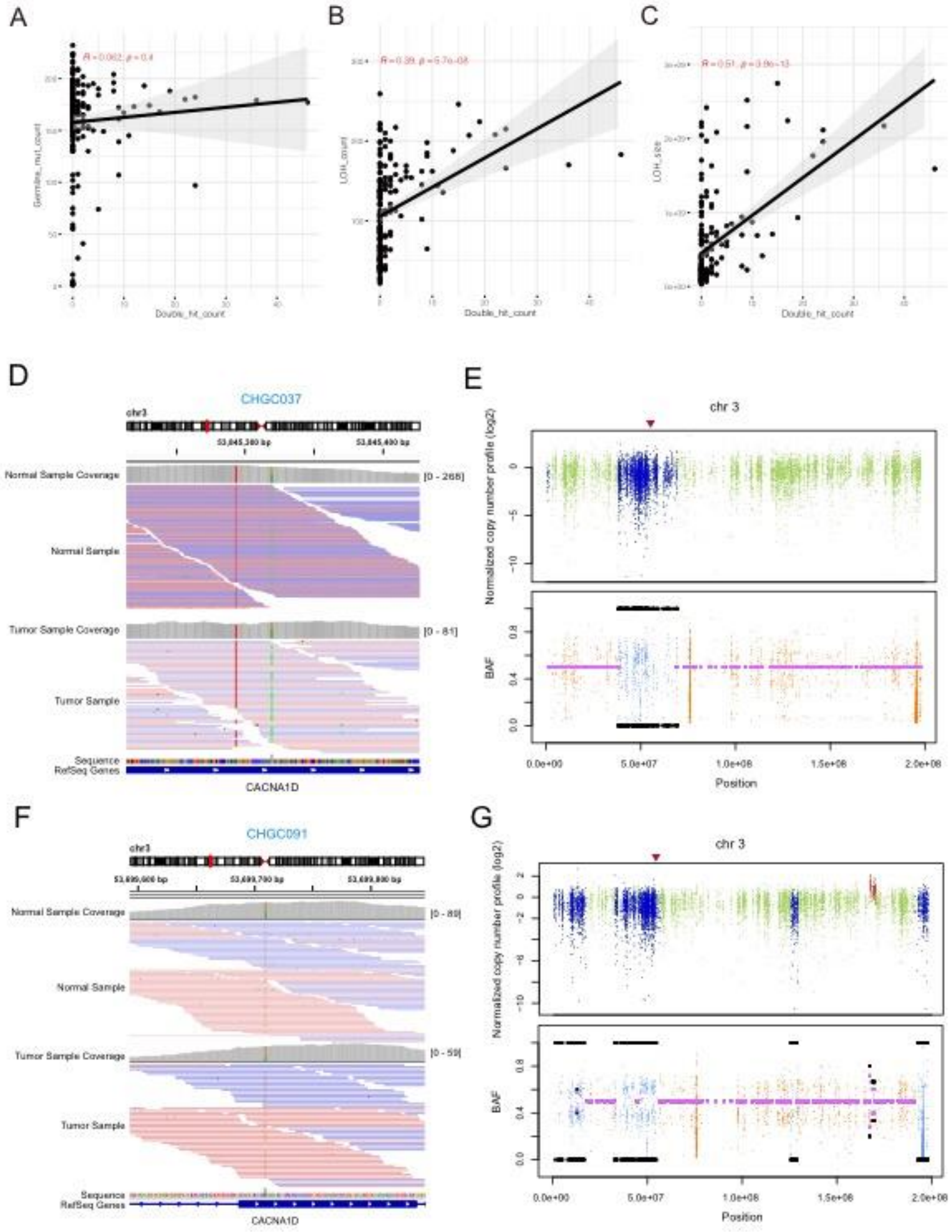

© 2022 Liu ZX et al. *JAMA Network Open*.

eFigure 9. Double-Hit Events Detected in COSMIC Cancer Census Genes from Patients With HDGC

A. *PREX2*. B. *CIC*. C. *COL1A1*. D. *GNA11*. E. *ZFHX3*. F. *CNOT3*. G. *NCOR1*. H. *FANCA*. I. *ARHGAP26*. J. *TRIP11*. K. *RNF213*. L. *CEBPA*. M. *GOLGA5*. N. *BRCA1*. Left: Reads mapping to the mutation point sites. Forward- and Reverse-mapping reads are colored in red and blue, respectively. Right: Copy-number variant and allelic imbalances of germline mutation sites. Copy-number variant profile is represented by log R ratio (LRR), centered at zero. Red points: amplificated regions; Blue points: deleted regions; Green points: neutral regions. Allelic imbalances are represented by B-allele frequency (BAF). Blue points: LOH regions; Red points: no-LOH regions. The germline mutation sites are marked by triangles.

© 2022 Liu ZX et al. *JAMA Network Open*.

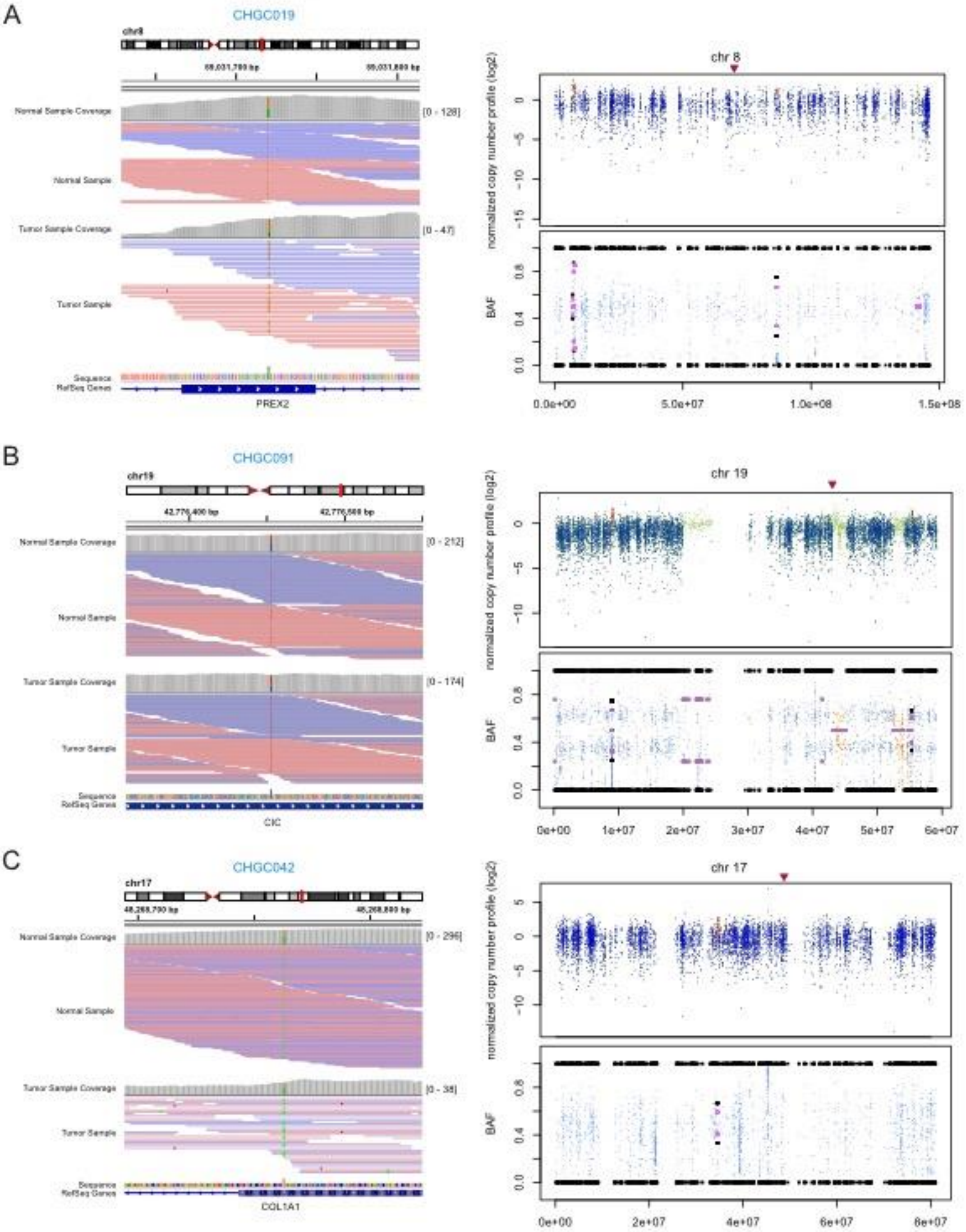

© 2022 Liu ZX et al. *JAMA Network Open*.

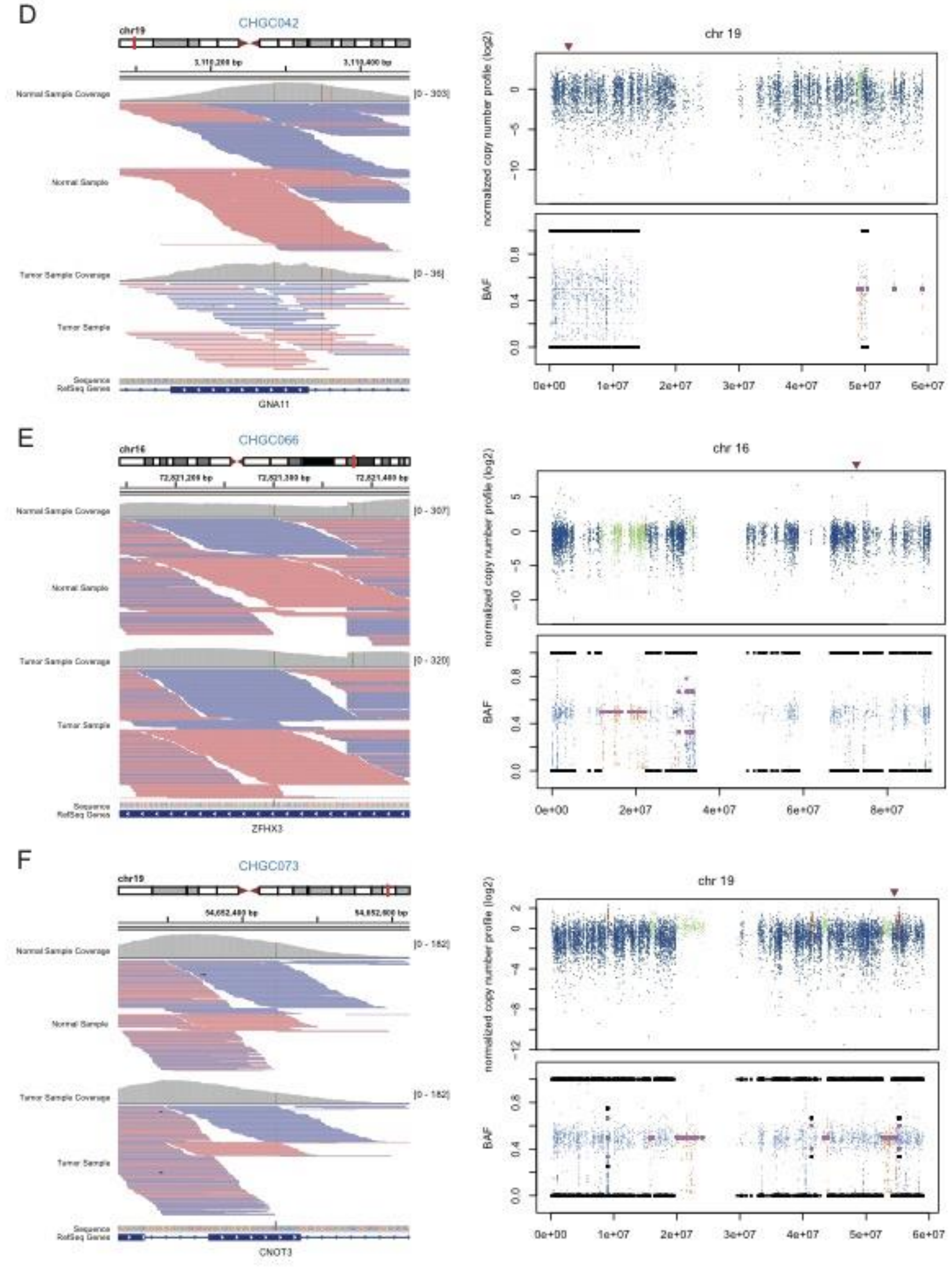

© 2022 Liu ZX et al. *JAMA Network Open*.

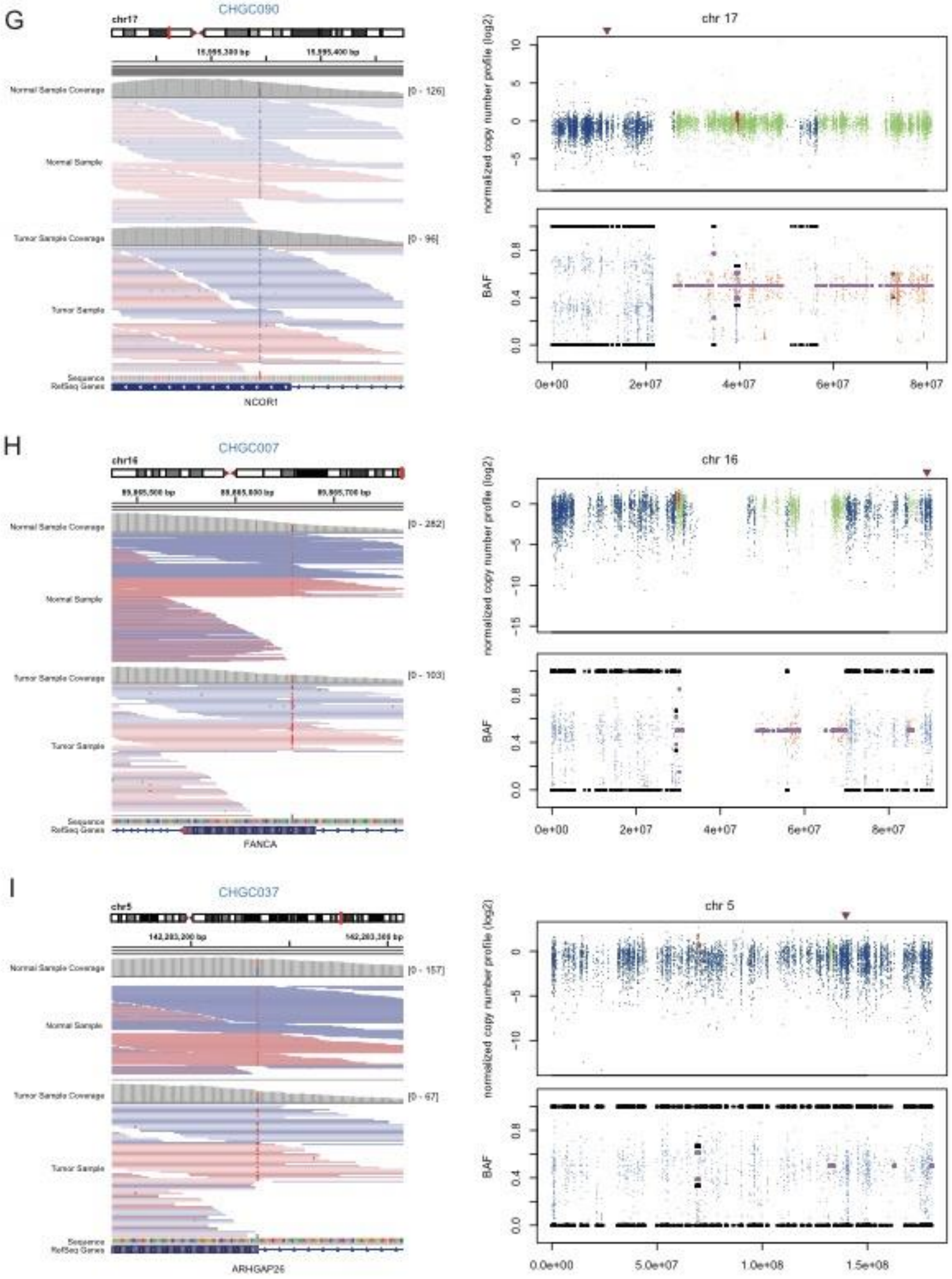

© 2022 Liu ZX et al. *JAMA Network Open*.

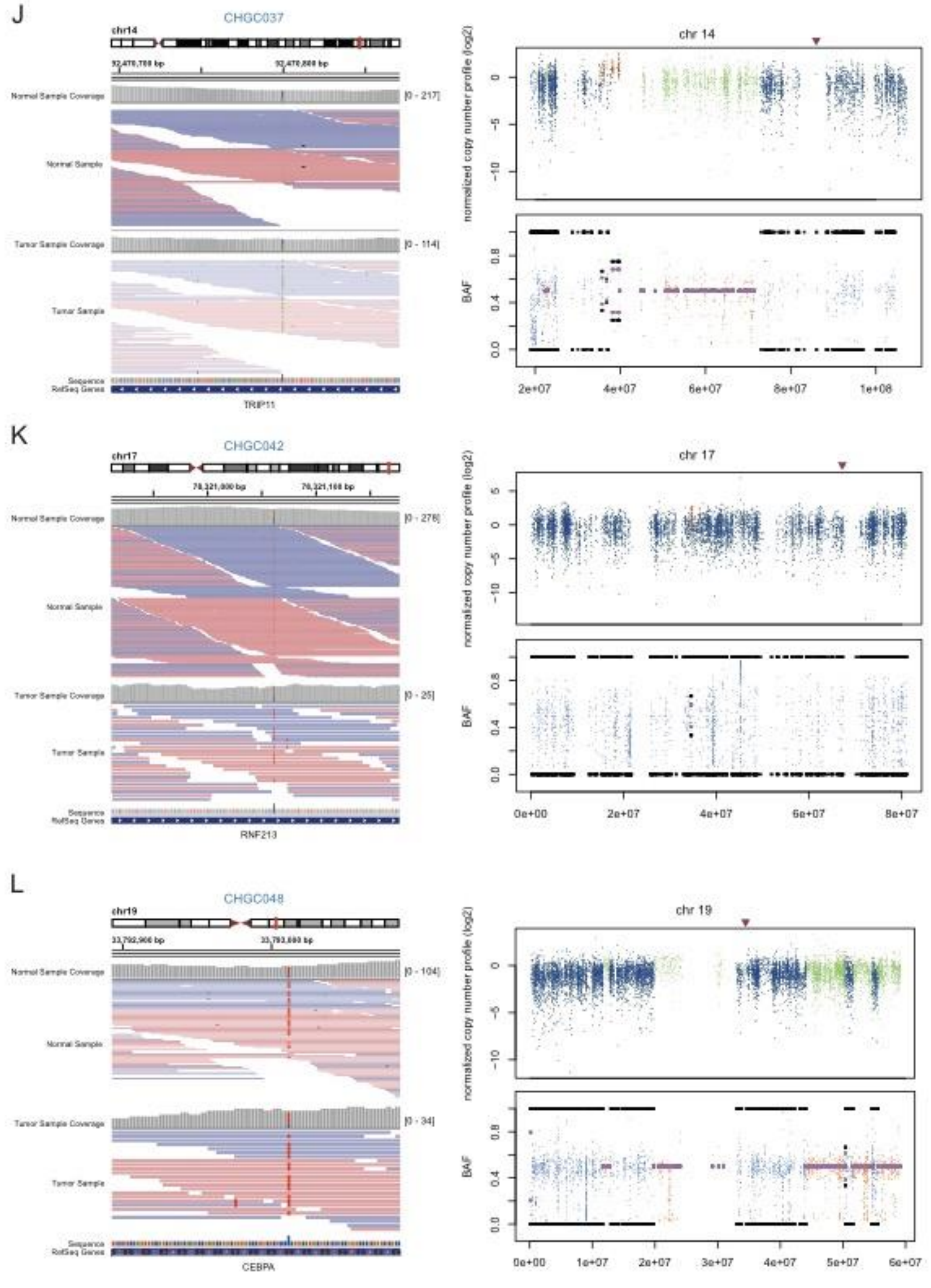

© 2022 Liu ZX et al. *JAMA Network Open*.

M

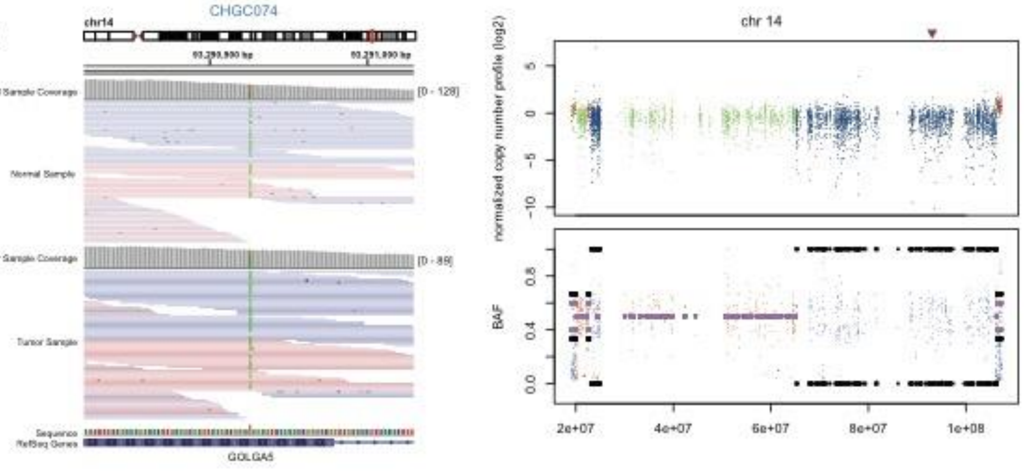

N

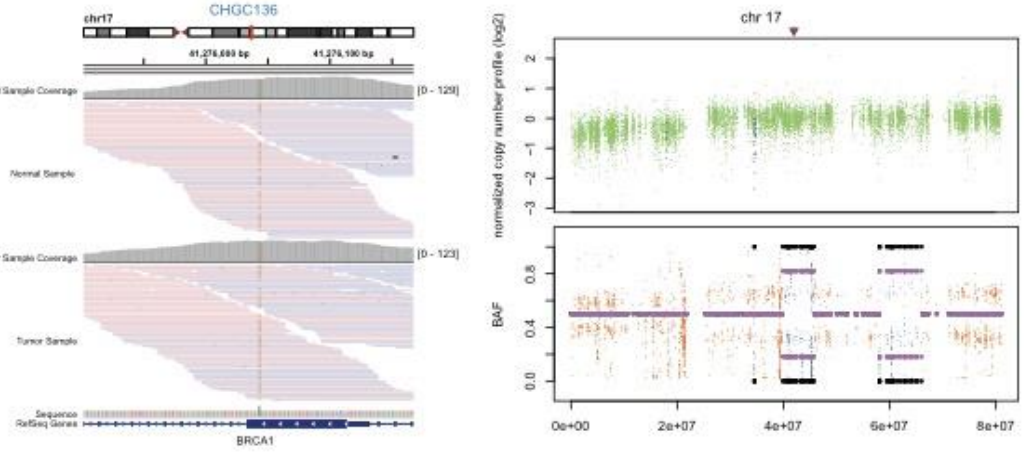

© 2022 Liu ZX et al. *JAMA Network Open*.

eFigure 10. Clinical-Associated Variants in HDGC

A. Genes with statistically significant association of non-silent germline mutation with over-all survival time (OS) in univariate Cox regression analysis stratified according to mutation frequency and univariate analysis p-value. Only genes with high mutation frequency (> 5%) are selected in this analysis and COSMIC cancer census genes with p-value < 0.05 (dashed lines) are highlighted. B-E. Kaplan-Meier survival curves for OS according to gene germline non-silent mutation status for patients with HDGC, including *MUC16* (B), *NCKAP5* (C), *HSPG2* (D) and *FSIP2* (E). F-H. Multivariate Cox regression analysis for the effect of non-silent germline mutations in *FSIP2* (F), *HSPG2* (G) and *NCKAP5* (H) combined with basic clinical information on OS of HDGC, respectively.

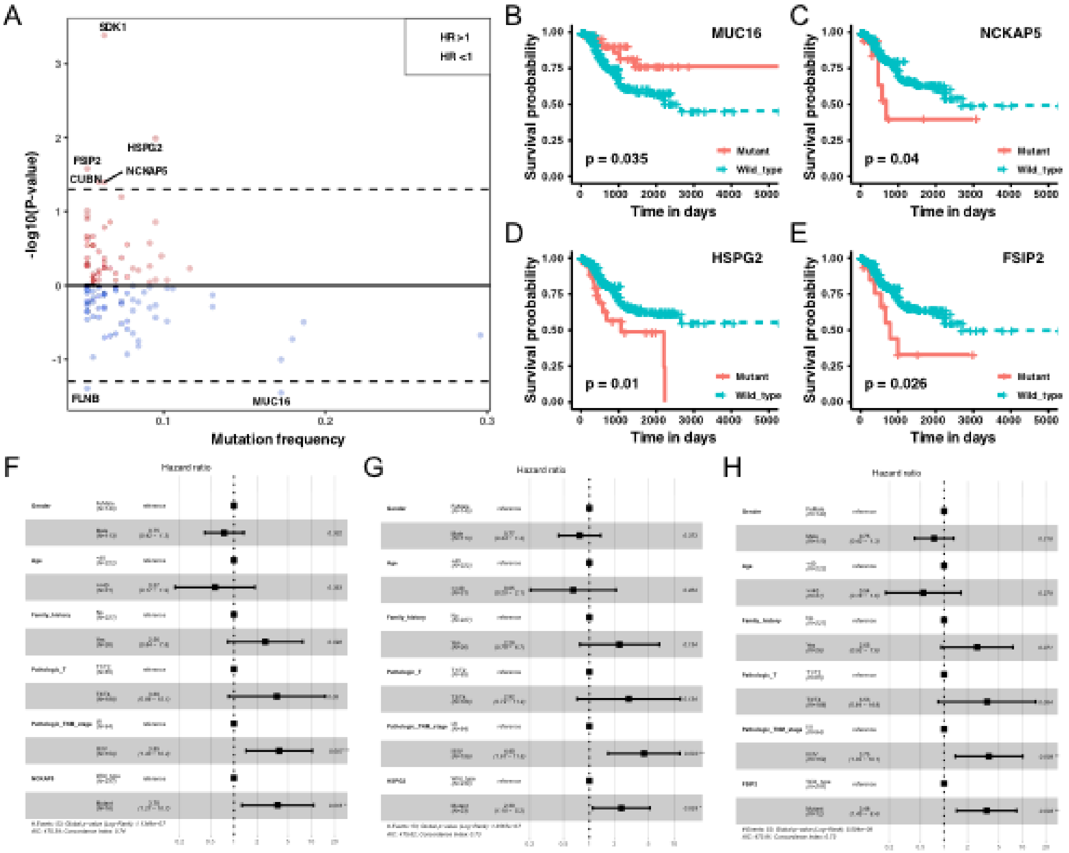

© 2022 Liu ZX et al. *JAMA Network Open*.

eFigure 11. Clinical-Associated Somatic Alterations (Nonsilent Alterations and CNAs) in HDGC

A. Genes with statistically significant association of somatic alterations with OS in univariate Cox regression analysis stratified according to alteration frequency and univariate analysis p-value. Only genes with high mutation frequency (> 5%) are selected in this analysis. COSMIC cancer census genes with alteration frequency > 10% (vertical dashed line) and p-value < 0.05 (horizontal dashed lines) are highlighted. B. Forest plots from multivariate Cox regression analysis shows the independent OS prognostic value of *FGFR3* from basic clinical information of HDGC. C-F. Multivariate Cox regression analysis for the effect of somatic alterations (including non-silent mutations and CNAs) in *ASPSCR1* (C), *CIC* (D), *DGCR8* (E) and *LZTR1* (F) combined with basic clinical information on OS of HDGC, respectively. G. Somatic alteration frequency of FGFR3 in HDGC cohort and TCGA cohort. H. Reactome pathway enrichment of genes in which somatic alteration are significantly associated with poor OS (Hazard ratio > 1). I. Reactome pathway enrichment of genes in which somatic alteration are significantly associated with longer OS (Hazard ratio < 1).

© 2022 Liu ZX et al. *JAMA Network Open*.

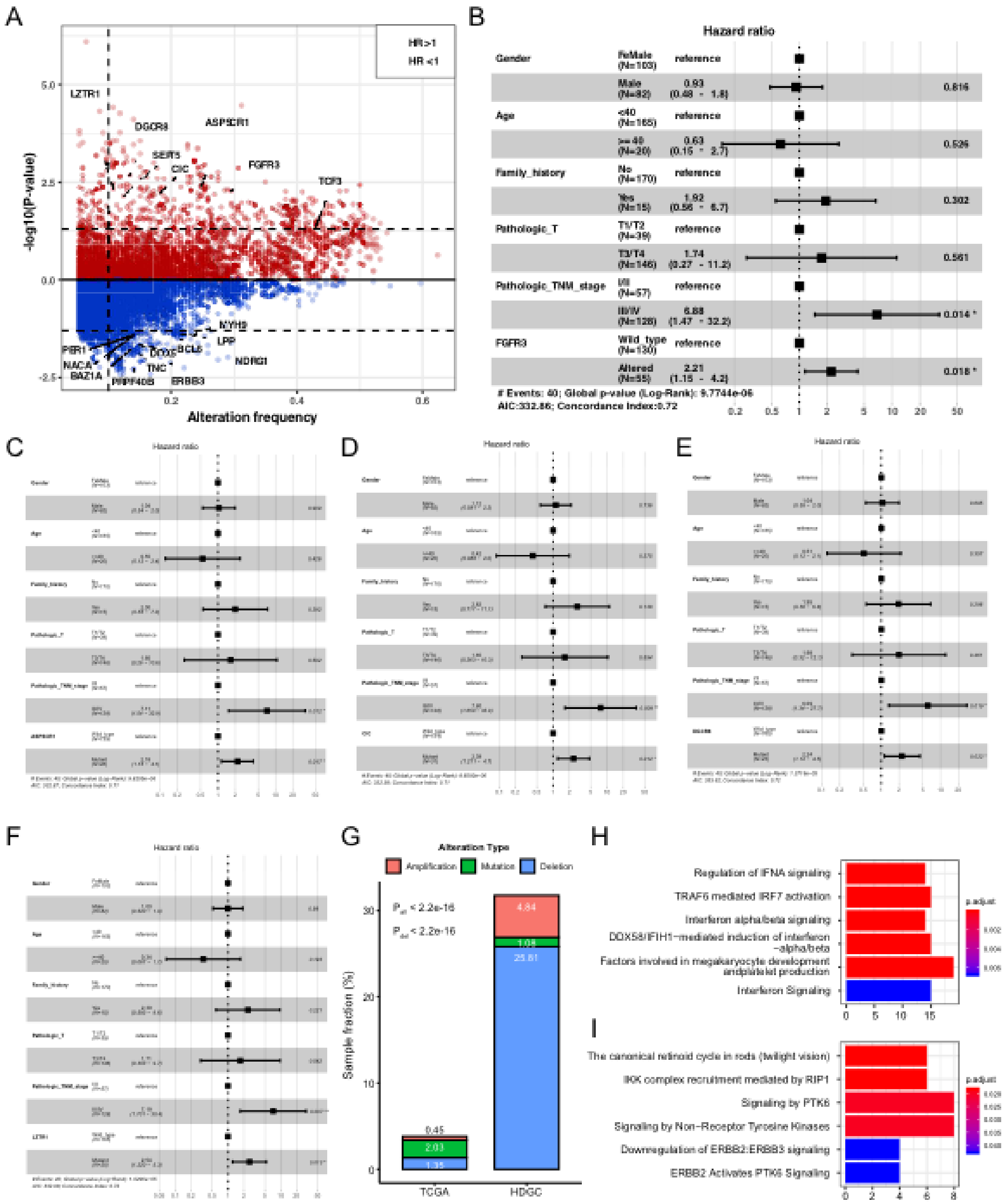

© 2022 Liu ZX et al. *JAMA Network Open*.

eFigure 12. Summary of Actionable Variants of HDGC

A. The bar graphs of the actionable somatic mutations. B. The bar graphs of the actionable somatic CNAs. ESMO Scale for Clinical Actionability of molecular Targets (ESCAT) tiers are shown separately.

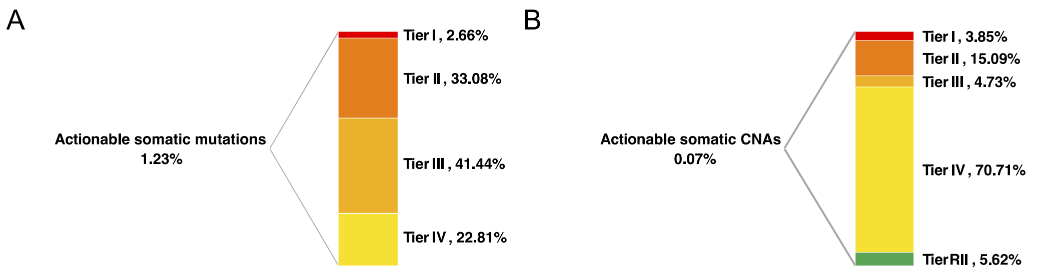

© 2022 Liu ZX et al. *JAMA Network Open*.

eReferences

1. Oliveira C, Pinheiro H, Figueiredo J, Seruca R, Carneiro F. Familial gastric cancer: genetic susceptibility, pathology, and implications for management. *Lancet Oncol.* 2015;16(2):e60-70.

2. Genomes Project C, Abecasis GR, Auton A, et al. An integrated map of genetic variation from 1,092 human genomes. *Nature.* 2012;491(7422):56-65.

3. Liu S, Huang S, Chen F, et al. Genomic Analyses from Non-invasive Prenatal Testing Reveal Genetic Associations, Patterns of Viral Infections, and Chinese Population History. *Cell.* 2018;175(2):347-359 e314.

4. Kent WJ. BLAT--the BLAST-like alignment tool. *Genome Res.* 2002;12(4):656-664.

5. Mayakonda A, Lin DC, Assenov Y, Plass C, Koeffler HP. Maftools: efficient and comprehensive analysis of somatic variants in cancer. *Genome research.* 2018;28(11):1747-1756.

6. Niu B, Ye K, Zhang Q, et al. MSIsensor: microsatellite instability detection using paired tumor-normal sequence data. *Bioinformatics.* 2014;30(7):1015-1016.

7. Chakravarty D, Gao J, Phillips SM, et al. OncoKB: A Precision Oncology Knowledge Base. *JCO precision oncology.* 2017:Jul;2017:PO.2017.00011.

8. Tamborero D, Rubio-Perez C, Deu-Pons J, et al. Cancer Genome Interpreter annotates the biological and clinical relevance of tumor alterations. *Genome Med.* 2018;10(1):25.

© 2022 Liu ZX et al. *JAMA Network Open*.

9. Griffith M, Spies NC, Krysiak K, et al. CIViC is a community knowledgebase for expert crowdsourcing the clinical interpretation of variants in cancer. *Nat Genet.* 2017;49(2):170-174.

10. Mateo J, Chakravarty D, Dienstmann R, et al. A framework to rank genomic alterations as targets for cancer precision medicine: the ESMO Scale for Clinical Actionability of molecular Targets (ESCAT). *Ann Oncol.* 2018;29(9):1895-1902.

11. Qiu MZ, He CY, Lu SX, et al. Prospective observation: Clinical utility of plasma Epstein-Barr virus DNA load in EBV-associated gastric carcinoma patients. *International journal of cancer.* 2020;146(1):272-280.

12. Qiu MZ, Cai MY, Zhang DS, et al. Clinicopathological characteristics and prognostic analysis of Lauren classification in gastric adenocarcinoma in China. *Journal of translational medicine.* 2013;11:58.

13. Roberts NJ, Norris AL, Petersen GM, et al. Whole Genome Sequencing Defines the Genetic Heterogeneity of Familial Pancreatic Cancer. *Cancer Discov.* 2016;6(2):166-175.

© 2022 Liu ZX et al. *JAMA Network Open*.
