# Supplementary material for: Whole-Exome Sequencing Among Chinese Patients With Hereditary Diffuse Gastric Cancer
Source: JAMA Netw Open. 2022 Dec 9;5(12):e2245836. doi: 10.1001/jamanetworkopen.2022.45836 (PMC9856492; doi:10.1001/jamanetworkopen.2022.45836)
Supplement: Supplement 3. — Data Sharing Statement [file jamanetwopen-e2245836-s003.pdf]

Data Sharing Statement

Liu. Whole-Exome Sequencing Among Chinese Patients With Hereditary Diffuse Gastric Cancer. *JAMA Netw Open*. Published December 09, 2022.
doi:10.1001/jamanetworkopen.2022.45836

Data
Data available: No

Additional Information
Explanation for why data not available: Currently the data could not be shared due to the genetic data privacy policy for the germline whole exome sequencing. We are applying the authorization from Human Genetics Resources Administration of China to release the data through Genome Sequence Archive for Human (https://ngdc.cncb.ac.cn/gsa-human/), but currently we have no timetable for the approval.
